# Microbial and diagenetic steps leading to the mineralisation of Great Salt Lake microbialites

**DOI:** 10.1038/srep31495

**Published:** 2016-08-16

**Authors:** Aurélie Pace, Raphaël Bourillot, Anthony Bouton, Emmanuelle Vennin, Serge Galaup, Irina Bundeleva, Patricia Patrier, Christophe Dupraz, Christophe Thomazo, Pierre Sansjofre, Yusuke Yokoyama, Michel Franceschi, Yannick Anguy, Léa Pigot, Aurélien Virgone, Pieter T. Visscher

**Affiliations:** 1 Ensegid Bordeaux INP, G&E, EA 4592, F-33600, Pessac, France; 2 Université Bordeaux Montaigne, G&E, EA 4592,F-33600, Pessac, France; 3 Laboratoire Biogéosciences UMR 6282 UBFC/CNRS, Univ. Bourgogne Franche-Comté, 6 boulevard Gabriel, Dijon 21000, France; 4 Université de Poitiers, UMR 6269 CNRS, HYDRASA, 40 avenue du Recteur Pineau, 86022 Poitiers, France; 5 Department of Geological Sciences, Stockholm University, Svante Arrhenius väg 8, Stockholm, 06269, Sweden; 6 Laboratoire Domaines Océaniques, Université de Bretagne Occidentale, UMR 6538 CNRS, 29280 Plouzané, France; 7 Atmosphere and Ocean Research Institute, Department of Earth and Planetary Sciences, University of Tokyo, 5-1-5 Kashiwanoha, Chiba 277-8564, Japan; 8 I2M, ENSAM, UMR 5285 CNRS, Esplanade des Arts et Métiers, 33405 Talence, France; 9 Total, CSTJF, Avenue Larribau, 64018 Pau, France; 10 Department of Marine Sciences, University of Connecticut, 1080 Shennecossett Road, Groton, CT 06340, USA

## Abstract

Microbialites are widespread in modern and fossil hypersaline environments, where they provide a unique sedimentary archive. Authigenic mineral precipitation in modern microbialites results from a complex interplay between microbial metabolisms, organic matrices and environmental parameters. Here, we combined mineralogical and microscopic analyses with measurements of metabolic activity in order to characterise the mineralisation of microbial mats forming microbialites in the Great Salt Lake (Utah, USA). Our results show that the mineralisation process takes place in three steps progressing along geochemical gradients produced through microbial activity. First, a poorly crystallized Mg-Si phase precipitates on alveolar extracellular organic matrix due to a rise of the pH in the zone of active oxygenic photosynthesis. Second, aragonite patches nucleate in close proximity to sulfate reduction hotspots, as a result of the degradation of cyanobacteria and extracellular organic matrix mediated by, among others, sulfate reducing bacteria. A final step consists of partial replacement of aragonite by dolomite, possibly in neutral to slightly acidic porewater. This might occur due to dissolution-precipitation reactions when the most recalcitrant part of the organic matrix is degraded. The mineralisation pathways proposed here provide pivotal insight for the interpretation of microbial processes in past hypersaline environments.

Microbial mats are metabolically diverse biofilm communities that potentially are the site for the precipitation of authigenic minerals, predominantly carbonates, but also silicates and oxides^1^. Following lithification, microbial mats form organo-sedimentary deposits referred to as microbialites^2^. Some of the microbialites in the Pilbara (Australia) date back to *ca*. 3.4 Ga and are evidence for the oldest known ecosystem^3^. Since the Archean, microbialites have colonized almost all environments, hence constitute a unique geological archive^4^. The mineralogy and geochemistry (e.g., trace elements concentrations, isotopic ratio) of microbialite minerals are used for the reconstruction of past Earth’s surface environmental conditions, e.g., oceanic redox conditions^5^,^6^, continental weathering^7^ or volcanic hydrothermal activity^8^.

The structure and composition of authigenic minerals in microbial mats depends on a complex interplay between extrinsic (physicochemical) and intrinsic (biological) factors^1^,^9^. Assessing the relative contribution of these factors is key to decipher the paleoenvironmental information recorded in ancient microbialites. The mineralisation mechanisms of ancient microbialites are inferred from comparisons with their modern counterparts. Microbial mats are abundant in hypersaline environments, e.g., lakes, salinas and lagoons^1^,^10^ where shell producing fauna are impoverished^11^. In the last decades, several mineralisation mechanisms have been proposed for modern hypersaline mats^12^,^13^,^14^. In seawater-fed lakes, several key microbial guilds are able to create steep chemical gradients^10^,^15^. Oxygenic photosynthesis is a key metabolism enhancing the nucleation of carbonate minerals by fixing CO_2_ and increasing pH^1^. Cyanobacteria produce copious amounts of extracellular organic matter (EOM), which provides the main site for mineral precipitation. EOM are composed of two pools: (i) high molecular weight organic carbon, notably extracellular polymeric substances (EPS) and (ii) low molecular weight organic carbon (LMWOC), mainly comprising ~C_1_-C_5_ compounds^16^. EPS provide structural integrity and allow the coexistence of diverse microbial communities in the mats^17^, whereas LMWOC is excreted by autotrophic microbes (e.g., cyanobacteria) to maintain a favorable internal redox potential and mostly serves as carbon source for heterotrophic metabolisms^16^. Acidic functional groups within the EOM, which deprotonate as a function of the pH^18^, bind cations (e.g., Mg^2+^, Ca^2+^) and thus strongly influence nucleation, structure and chemical composition of carbonate minerals^15^,^17^,^18^. Precipitation will only take place through alteration of the ion-binding EOM matrix either through physicochemical (e.g., extrinsic supersaturation, UV degradation) or microbial (e.g., heterotrophic degradation releasing cations) processes^1^,^14^.

A few studies have shown the precipitation of poorly crystallized magnesium silicates (kerolite, stevensite; named here Mg-Si phases) on EOM and cell material in both seawater-fed and continental lakes^19^,^20^. Three chemical conditions are required for Mg-Si phases to precipitate: an elevated pH (>8.6–8.7) combined with high dissolved silica and magnesium activities^20^,^21^. These Mg-Si minerals also participate to the fossilisation of microorganisms (e.g., cyanobacteria, diatoms) through permineralisation. Due to their intimate link with organic matrices, these minerals could potentially include organic molecules and help their preservation through diagenesis^20^. However, it is still unknown if microbial activity has a direct or indirect role in the precipitation of these minerals, and if so, which metabolisms are involved. Mg-Si phases are also associated with carbonate minerals (e.g., aragonite, hydromagnesite), which should theoretically precipitate under different chemical conditions. Therefore, establishing the sequence of their precipitation (paragenesis) could help to interpret the evolution of microbial and chemical reactions in the mat.

The margins of Great Salt Lake (GSL) are extensively covered by microbial mats and microbialites^22^. GSL, the fourth largest endorheic lake in the world is a hypersaline (salinity 50–285 g.l^−1^; average 120 g.l^−1^ since 1876), shallow system (depth <10 m) covering an average area of 4480 km^2^ 23,24. Due to its size and water chemistry, GSL is considered as an analogue to some ancient large scale hypersaline lakes^25^, and its mineral composition has been studied for the detection of paleolakes in future Mars missions^26^. In addition to carbonate minerals, Eardley^27^ identified hydrated magnesium silicates in GSL microbialites. He postulated that these Mg clays were chemically precipitated in the water column and trapped in microbial mucus. Eardley was the first to suggest a role of cyanobacteria in carbonate precipitation in GSL.

In this multidisciplinary study, we assessed the structure, composition and sequential precipitation of mineral phases, including Mg silicates, in GSL microbial mats and microbialites. The processes of mineralisation were investigated by comparing the mineral paragenesis with measures of microbial activity. The conceptual mineralisation model proposed here provides a context for the interpretation of the geochemical and mineral composition of fossil microbialites.

## Results

**Figure 1:**
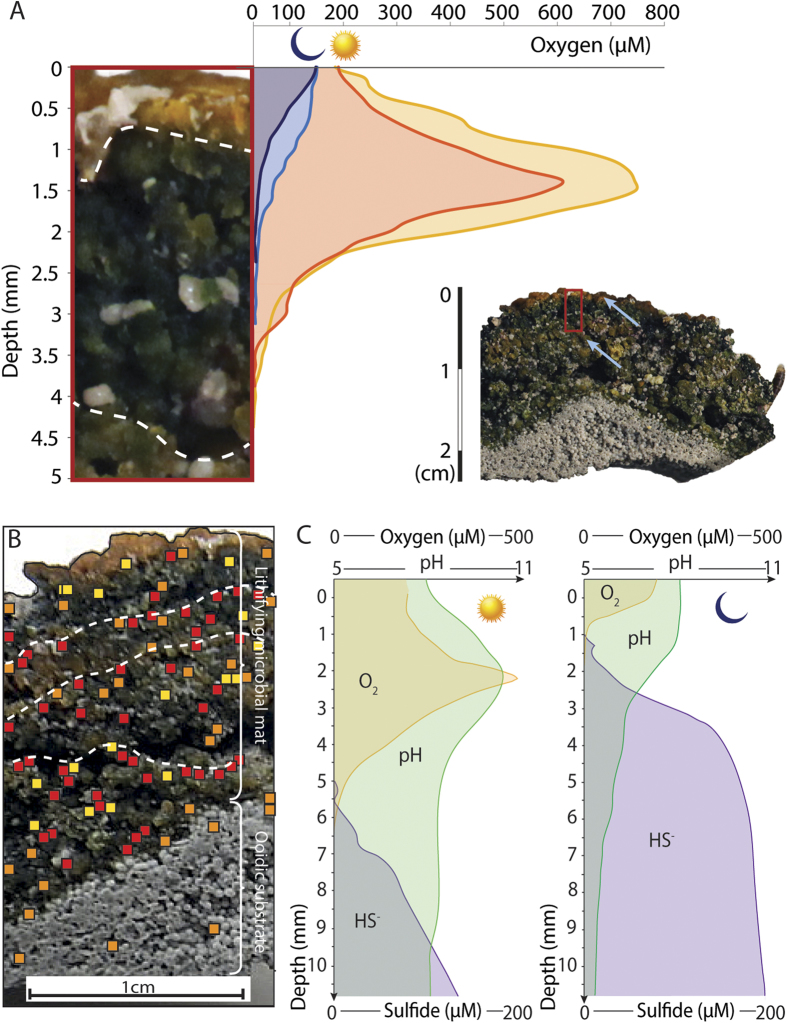
Microbial activity in lithifying mats of GSL. (**A**) Representative O_2_ depth profiles of the top of the mat (red rectangle in hand sample image insert), measured in daylight (red and orange curves) and during the night (light and dark blue curves). The peak of O_2_ production indicates oxygenic photosynthesis is maximum at *ca*. 1.5 mm (**B**). Distribution of sulfate reduction determined on the identical sample as used for the oxygen profiles (panel A). Red, orange and yellow squares correspond respectively to high, medium and low sulfate reduction rates. (**C**) Representative O_2_, pH and HS^−^ depth profiles during day (left) and night (right). See text for detail.

Two types of microbial structures, modern lithifying microbial mats and fossil microbialites (Age 2.8 Ka calBP; Supplementary Figs S1 and S2 and Supplementary Table S1), were sampled along the shore of Antelope Island and offshore Eardley Spit (Supplementary Fig. S1). The lithifying mats showed a succession of thin orange and thick porous green layers The orange color is indicative of carotenoids, protecting the deeper part of the mats by quenching UV^28^. This implies that the orange layers could represent the former surfaces of the mat. Green and orange layers were composed of contorted *ca*. 0.3 to 2 mm clusters of coccoid cyanobacteria (Fig. 1; Supplementary Figs S3 to S6). These clusters compose the bulk of the mat framework, and remain quite similar in shape and composition from the base to the top. Two dominant coccoid morphotypes were recognized inside clusters (Supplementary Fig. S4). Pennate diatoms were also observed on the surface of the mats (Supplementary Fig. S7A). The maximum of oxygen production during daylight (600–800 μM) occurred at *ca*. 2 mm depth, in the uppermost green lamina (Fig. 1A). This subsurface oxygen peak resulted from active photosynthesis, predominantly by coccoid cyanobacteria. The oxygen level rapidly decreased below 2 mm. The concave shape of the curve indicates oxygen consumption, probably by heterotrophic bacteria. The sulfide concentration increased with depth and reached values of 130 and 185 μm at 11 mm during the daytime and nighttime respectively (Fig. 1C). The daytime pH value peaked at 10.4 in close proximity to the O_2_ maximum. In the anoxic part of the mat, at the end of the nighttime, the pH had declined to ca. 5.4, possibly due to accumulation of fermentation products^1^. Active sulfate reduction spots were found throughout the mat, including a few spots in the substratum, where the mat penetrated locally (Fig. 1B). There was a marked spatial concurrence between sulfate reduction (SRA) hotspots and aragonite-dominated patches (0.1–2 mm in diameter; Fig. 1B; Supplementary Fig. S8; Supplementary Table S2): aragonite was observed at 97.8% of spots with high SRA, 64.5% of spots with medium SRA and 82.3% of spots with low SRA.

**Figure 2:**
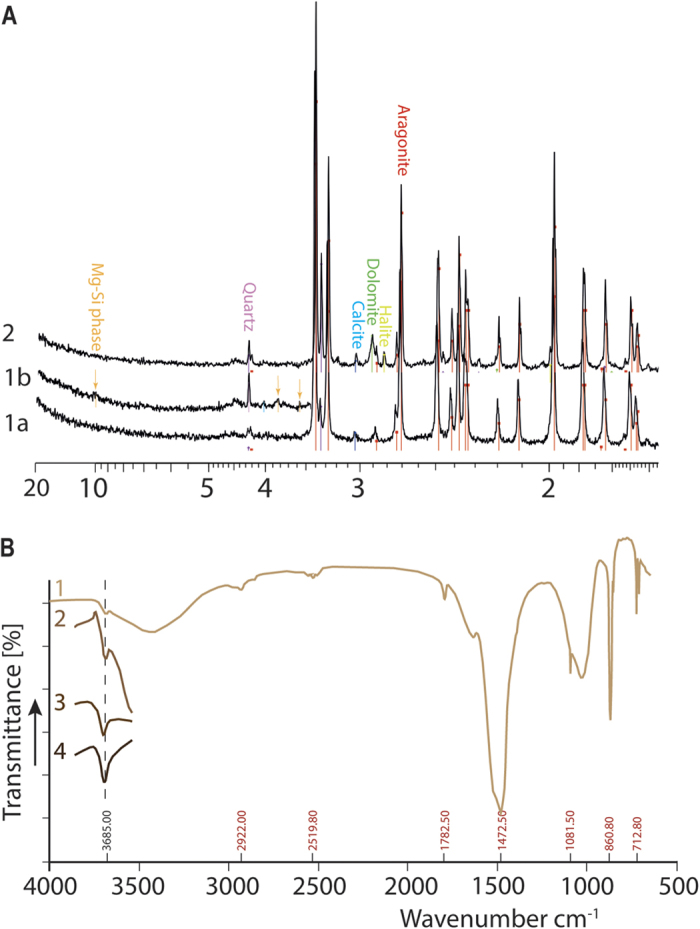
(**A**) X-ray diffraction (XRD)-based composition of the mineral fractions from a lithifying mat (Curve 1a: fraction <100 μm; Curve 1b: fraction < 2 μm) and a microbialite (Curve 2; bulk). (**B**) Fourier Transform Infrared Spectrometry (FTIR) of a lithifying microbial mat (1). The absorption band at 3685 cm^−1^ indicates the presence of trioctahedral Mg_3_-OH groups. The others bands (red values) are typical of aragonite. Curves 2, 3 and 4 show the evolution of the 3685 cm^−1^, band at respectively 150 °C, 200 °C and 300 °C.

In both lithifying mats and microbialites, carbonate patches fused to form an intestine-like clotted thrombolite fabric (Supplementary Figs S2 and S3). The lower part of the microbialites showed a laminated structure (stromatolitic; Fig. S2F). The X-ray diffraction patterns (Fig. 2A) showed that the predominant minerals were aragonite (CaCO_3_) and quartz, and in addition that microbialites included dolomite [CaMg(CO_3_)_2_].

**Figure 3:**
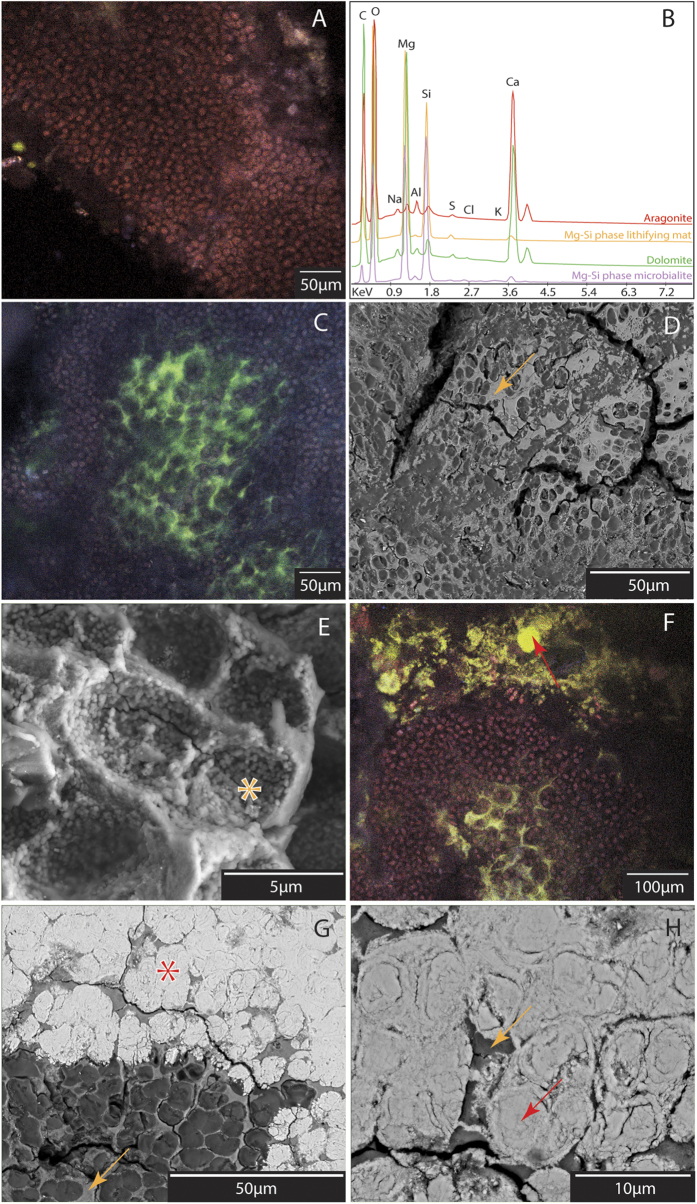
Characterisation of the successive mineralisation steps and composition of the mineral phases in a lithifying mat. (**A**) CLSM image of photosynthetically active coccoid cyanobacteria (red fluorescence) embedded in extracellular organic matrix (EOM). (**B**) Representative EDS spectra of the four main mineral phases, each color corresponding to a specific mineral. The location of EDS analyses is indicated by asterisks on the SEM images. (**C**) CLSM image showing photosynthetically active coccoid cyanobacteria (red) embedded in EOM. The green fluorescence labelling is indicative of binding of Mg by the EOM. (**D**) SEM image of the same sample as C (CLSM). EOM is completely covered in Mg-Si anhedral, round crystals (orange arrow). (**E**) Image showing EOM casts of coccoid bacteria, covered by round Mg-Si crystals (Mg-Si phase lithifying mat; orange asterisk; EDS signature Fig. 3B and Supplementary Table S3). (**F**) CLSM image showing aragonite (bright yellow fluorescence; red arrow; corresponding raman spectrum can be found in Supplementary Fig. S9) nucleating in zone lacking photosynthetically active cyanobacteria. (**G**,**H**) SEM images showing aragonite nucleation (Ar; red asterisk locates the EDS spectrum Fig. 3B). Aragonite (AR) precipitates first inside dead coccoid cells, mineralises their wall and expands into the surrounding EOM, partly covered by Mg-Si phase (orange arrow).

Observations using confocal laser scanning microscopy (CLSM) and scanning electron microscopy (SEM) showed a recurrent mineral superposition (Fig. 3; Supplementary Figs S3,S5,S6 and S7). In the lithifying mat, photosynthetically active coccoid cyanobacteria (Fig. 3A) were embedded in alveolar EOM (Supplementary Fig. S7B and S7C). The calcein labelling (Fig. 3C and Supplementary Fig. S4A) indicates local enrichments of these EOM in Ca^2+^ or Mg^2+^. SEM images of the same zones (Fig. 3D,E) showed the EOM completely covered by anhedral, round, *ca*. 200 nm, Mg-Si rich crystals (Fig. 3B; Supplementary. Fig. S3). Some other parts of the coccoid clusters -including zones with dense EOM- were devoid of mineral precipitation. FTIR analyses (Fig. 2B) of the same sample revealed an absorption band at 3685 cm^−1^, indicating the presence of trioctaedral Mg_3_-OH groups, typical of the sepiolite or stevensite mineral groups^29^. Successive FTIR analyses on heated powders (150 to 300 °C) showed a progressive increase of this peak associated with the dehydration of the sample. Two small X-ray diffraction peaks (respectively at 4.506 and 10.03 Å) were observed in the fraction <2 μm of some samples (Fig. 2A), confirming the identification of Mg-silicates. The calculated structural formula (Supplementary Table S3) pointed to an excess of Mg and a deficit of Si compared to both standard sepiolite [Mg_4_Si_6_O_15_(OH)_2_.6(H_2_O)] and stevensite [(Ca_0.5_,Na)_x_(Mg,Fe^2+^)_3-x_Si_4_O_10_(OH)_2_ n(H_2_O)].

**Figure 4:**
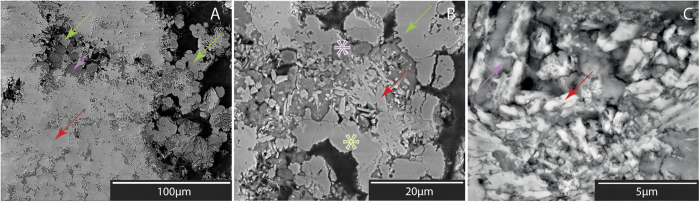
SEM images of a fossil microbialite. (**A**,**B**) ‘Rice grain’ aragonite crystals (red arrow) are covered by patches of Mg-Si crystals (Mg-Si phase microbialite; purple asterisk; EDS signature Fig. 3B and Supplementary Table 3). Dolomite precipitates (green asterisk and arrows; EDS signature Fig. 3B) located at the edge of the around and inside aragonite (red arrow) patches. (**C**) Aragonite crystals showing irregular borders, holes indicating dissolution.

Macroscopically, intestine-like aragonite perfectly mimicked the patchy framework composed by coccoid clusters (Supplementary Fig. S4D, S4E and S4F). The zones of aragonite precipitation were devoid of photosynthetic pigments (Fig. 3F and Supplementary Fig. S9). SEM images showed aragonite crystals, nucleating first inside coccoids, then permineralising their wall (Supplementary Fig. S6). Then, aragonite expanded into the surrounding alveolar organic matrix precipitating around and embedding the Mg-Si phase crystals –as shown by the increase in Mg, Si and Al from the inside to the outside of the cell (Supplementary Fig. S5)– and finally replaced most of the surrounding organic framework (Fig. 3B,G,H). The same pattern was observed at all spots of aragonite precipitation in the mat, regardless of the depth. In the microbialite (Fig. 4A and Supplementary Fig. S2F), patches of aragonite embedding Mg-Si round crystals were still abundant. The Mg-Si phase showed a relative increase in Si and decrease in Mg compared to the lithifying mat (Supplementary Table S3). The structural formula that was calculated appeared closer to that of stevensite or sepiolite than in the lithifying mat. Spherical aggregates (5–10 μm in diameter) of quasi-stoichiometric dolomite micro-rhombs (50.5 mol% of MgCO_3_) developed around and inside aragonite-Mg-Si phase patches (Figs 3B and 4A,B, and Supplementary Fig. S10). These aggregates were locally surrounded by alveolar EOM, and were similar in shape and size to coccoid cyanobacteria (Fig. 4B and Supplementary Fig. S7G and S7H). They locally developed inside dissolution micropores within aragonite crystals (Fig. 4C).

### Mineralisation model

**Figure 5:**
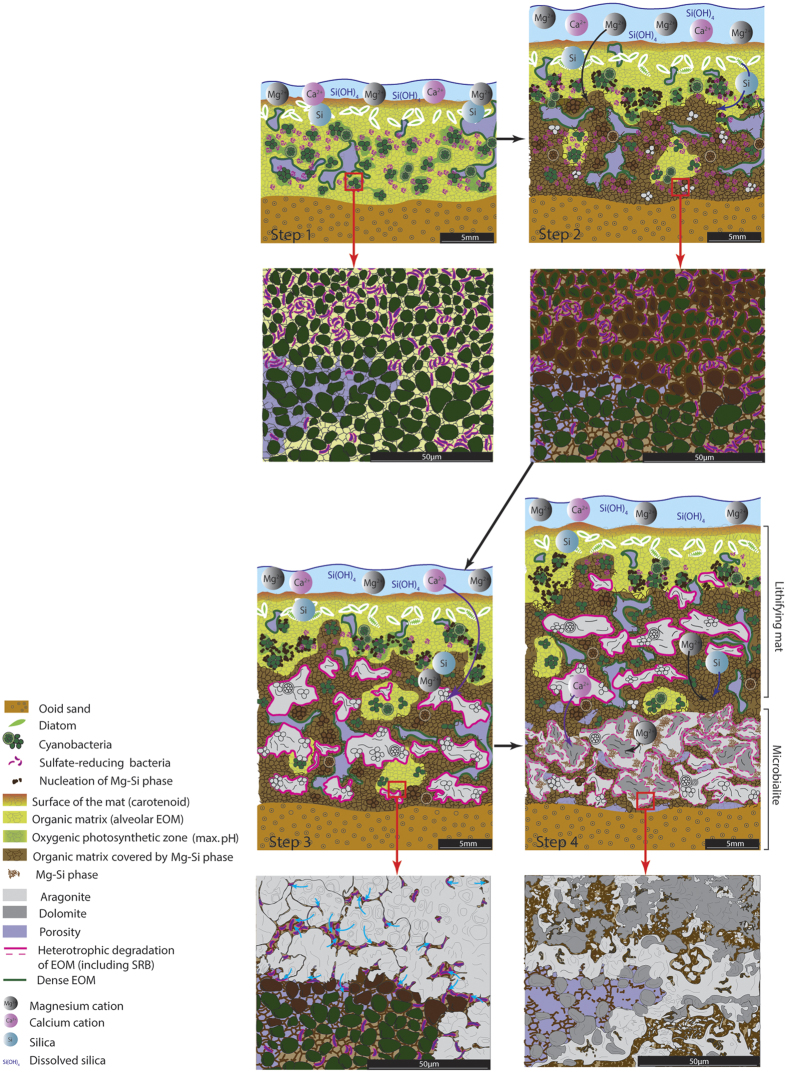
Conceptual model of microbialite mineralisation in GSL (see text for detail). Step 1: Development of a microbial mat on a hard substrate. Step 2: Mg-Si phase nucleation on the EOM in the zone of active oxygenic photosynthesis. Two sources of silica can be identified: lake water-dissolved silica and silica originating from the dissolution of diatom skeletons in the zone of maximum photosynthesis (pH > 10). Step 3: Degradation of EOM by heterotrophs, notably SRB, allowing the nucleation of aragonite. Aragonite precipitates first inside dead coccoid cells and then extends into the EOM (blue arrows). Degradation of EOM creates a zone of low pH in the deepest parts of the mat, inducing a partial dissolution of aragonite. Step 4: Pockets of partially degraded EOM bind Mg^2+^ (from porewater and possibly from Mg-Si phase) and Ca^2+^ (from aragonite dissolution), leading to the nucleation of dolomite at the interface between degraded EOM and previously precipitated aragonite/Mg-Si phase patches.

Based on the mineral sequence we observed, a four steps scenario leading to the lithification of GSL microbialites emerges (Fig. 5):

(i) During the initial phase, a microbial mat dominated by clusters of coccoid cyanobacteria (Fig. 3A and Supplementary Figs S3, S4B and S4C) develops on the lake floor (Fig. 5 *step1*).

(ii) Inside coccoid clusters, the cation-binding alveolar EOM constitute the site for nucleation and mineral growth of a Mg-Si phase (Supplementary Fig. S3; Fig. 5 *step2*). GSL surface water is supersaturated for the main Mg-silicate minerals (Supplementary Fig. S11 and Supplementary Table S4). However, its pH (*ca*. 8.2) is below the threshold (pH> 8.6–8.7) above which Mg silicates precipitation is experimentally observed^21^. In contrast, high rates of photosynthesis yield pH values above 10, thereby favoring the nucleation of the Mg-Si phase in the uppermost millimetres of the mat. Geochemical modeling indicates that at pH > 9, amorphous silica would dissolve in GSL (Supplementary Fig. S12). The dissolution of diatoms skeleton inside the mat (opal A; [SiO_2_, nH_2_O]) represents an additional source of dissolved silica, which could be incorporated in the Mg-Si phases.

(iii) During the third step (Fig. 5 *step3*), aragonite nucleates and grow in pockets created by the microbial degradation of coccoid cells and EOM. The aragonite nucleates first inside coccoid cells, then permineralises bacterial walls and finally precipitates around the Mg-Si crystals covering the EOM (Fig. 3G,H; Supplementary Fig. S6). This precipitation is strongly correlated with ‘hot-spots’ of sulfate reduction activity (Fig. 1B), which is indicative of a critical role of SRB in the organomineralisation process. As demonstrated in seawater-fed lakes and open marine environment, SRB are known to utilize the LMWOC fraction of the EOM^16^,^30^ producing (i) dissolved inorganic carbon increasing alkalinity and, (ii) sites for carbonate nucleation on the EPS matrix^14^,^31^. This process leads to intimate complexes of organic matter and minerals within the EOM where active sulfate reduction takes place. Consequently, aragonite precipitation could result from LWMOC consumption by SRB in both oxygenic and anoxygenic zones of the mat^32^. Aragonite will progressively replace the EOM, participating to the lithification of the mat.

(iv) The final step (Fig. 5 *step4*) characterises the transition from the lithifying mat to the microbialite. From step 3 to step 4, the Mg-Si phase evolves chemically, with a decreasing Mg:Si ratio (Supplementary Table S3). Aragonite is partially dissolved (Fig. 4C) and dolomite precipitates at the interface between EOM and aragonite-Mg-Si phase patches. Ca^2+^ supplied by aragonite dissolution could be incorporated into dolomite, and, similarly, Mg^2+^ from release by the Mg-Si phase and what remained in the porewater. Significant pH variations were documented during diel cycles in GSL microbial mats (Fig. 1C). During the day, pH decreases to 7.9–8.3 (i.e., the pH of the lake water) below the photosynthetically active zone. At the end of the night, the pH becomes slightly acidic in the permanently anoxic part of the mat, which we attribute to accumulation of fermentation products. Chemical modelling shows that at pH range from 6.5 to 7.5, i.e. values which prevail in the deeper layers during most of the day, aragonite would thermodynamically dissolve, whereas dolomite would still be oversaturated (Supplementary Fig. S12).

In spite of supersaturation, dolomite was not detected in GSL lithifying mats. Dolomite nucleation is known to be inhibited at ambient pressure and temperature in inorganic medium^33^. Various experiments have documented that this inhibition is alleviated when specific organic substrates are involved in the precipitation^34^,^35^,^36^. Carboxyl functional groups are particularly efficient in binding metallic cations and can promote dolomite nucleation by removing the hydration shell of Mg^2+^ ions, even in the absence of active microbial metabolism^36^. In GSL microbialites, the stable carbon isotope composition (δ^13^C = 3.06‰) of the dolomite (SI Materials and Methods, Supplementary Fig. S13 and Table S1) is *ca*. 2‰ less than the co-occurring aragonite patches (δ^13^C = 4.98‰). The microbial degradation of organic matter (including EOM) lowers pH, thereby modifying the composition of dissolved inorganic carbon (DIC) and inducing a negative shift in the δ^13^C of DIC^37^. The carbonates precipitated in microbial mats from this DIC would have more negative values^38^. We postulate that in GSL microbialites, dolomite precipitates following EOM degradation during long-term exposure to porewater with circum neutral pH values.

## Discussion

### Comparison with other lakes

The paragenesis in GSL microbialites reflects a pronounced modification of the porewater physicochemistry (e.g., dissolved oxygen, pH) through changes of microbial metabolisms, some of which strongly depend on the location within microbialite (i. e., depth-dependent, such as oxygenic photosynthesis).

Poorly crystallized Mg-Si phases, e.g., stevensite (smectite mineral group), kerolite and sepiolite (sepiolite mineral group), are now known to be major authigenic components of microbialites in modern saline and alkaline lakes^19^,^20^,^39^. These phases have been observed in lakes both with a pH above (Mexican crater lakes)^20^ or below^19^ the 8.6–8.7 threshold required for their precipitation. Consequently, physicochemical processes alone cannot explain the precipitation of these minerals. In GSL, the observed Mg-Si phase, likely a precursor of stevensite or sepiolite, is precipitated on the organic matrix by incorporating water Mg^2+^ and dissolved silica originating from lake water (Supplementary Fig. S13 and Supplementary Table S4) and potentially from diatom dissolution. Active oxygenic photosynthesis, promoting elevated pH conditions by producing OH^−^, seems to be the microbial mechanism inducing the precipitation of this poorly crystallized phase. Here, microbial activity allows for lowering the kinetic barrier for Mg-Si phase nucleation. In addition, laboratory precipitation experiments demonstrate that some organic compounds that make up the EOM pool enhance Mg-Si phase nucleation (e.g., succinic acid), whereas others inhibit this (e.g., oxalic acid). Succinic acid also seems to influence the formation of dioctahedral (Mg-rich) vs. trioctahedral (Al-rich) smectites^40^. Future research will focus on the influence of various natural EOM components on the composition of Mg-Si phases.

The precipitation of aragonite in GSL microbial mats is probably related to degradation of EOM and cell material by a microbial community in which SRB play an important role. Open marine stromatolites and thrombolites in Bahamas show similar examples of aragonite precipitation at sulfate reduction hotspots^15^,^32^,^41^,^42^. The Mg-Si phase-aragonite parasequence has been observed in microbialites from other alkaline lakes, including Mono Lake tufas (USA)^39^ and Lake Clifton thrombolites (Australia)^19^. In Lake Clifton, stevensite nucleates on the organic matrix and around cyanobacterial cells, with aragonite subsequently nucleating first inside cyanobacteria cells or impregnating their wall, and then covering the organic matrix and the cells. Contrastingly, in microbialites from lake Alchichica (Mexico), needles of aragonite nucleate first on the EOM, then impregnate the cell wall, the last step consisting in prismatic aragonite infilling the cell^43^. No authigenic microbial Mg-Si phase was documented in this lake with low dissolved silica (Si < 1.5 mg.l^−1^)^44^. The presence/absence of a Mg-Si phase seems critical for the nucleation and the development of aragonite crystals. By encapsulating EOM, the Mg-Si phase would slow their degradation by heterotrophic bacteria. Cell material (cytoplasm and cell wall) would thus be easier to degrade, thereby constituting the first locus of aragonite precipitation. Our observations are in agreement with Zeyen *et al*.^20^, who demonstrated that organic carbon -including possibly EOM- was intimately linked with kerolite and suggested that Mg silicates could incorporate and preserve biomarkers.

### Implications for the fossil record

The preservation and/or modification of the primary microbial microstructures as well as the mineral sequence after diagenesis are critical when interpreting paleoenvironmental conditions and/or microbial metabolisms from fossil microbialites. Mg-Si phases have been found associated with microbialites in hypersaline paleolakes. For example, thrombolitic columns formed in the high energy littoral zones of hypersaline and alkaline lake deposits found in the Eocene Green River Formation (54–43 Ma)^45^. Some of these buildups show a similar fabric to GSL microbialites, which suggests that they could have formed through comparable processes. The presence of intestine-like structures in the Green River thrombolites^46^ could indicate heterotrophic EOM degradation. The clay mineral composition of these ancient microbial buildups was not reported, but stevensite was found in the ooids surrounding microbialites^47^. Stevensite and talc have also been reported in deeply buried microbialites and oncoids of the early Cretaceous (Barremian) rift deposits offshore in the Congo and Cabinda basins^48^.

Zeyen *et al*.^20^, postulated that authigenic microbial Mg-Si phases have a great potential for the preservation of organic biomarkers. This implies to understand their evolution through diagenesis. Mineral transformations have been observed in laboratory experiments reproducing burial and subsequent diagenetic conditions: after a few weeks under hydrothermal conditions, poorly crystallized Mg-Si phases tend to recrystallize as kerolite (T = 180 °C), and eventually as more stable minerals, such as talc, especially under increased temperature and pressure (T = 400 °C; 1kbar)^21^. Similarly, at high temperature (T = 200–300 °C), sepiolite is also converted to stevensite^49^. Tosca *et al*.^21^ found talc, probably derived from a poorly crystallized Mg-Si phase, in association with dolomite in Neoproterozoic microbialites (800–700 Ma) from Yukon and Svalbard. Based on laboratory experiments, these authors explain the formation of the precursor of this talc by an increase in porewater pH resulting from anaerobic respiration and bacterial sulfate reduction. The current investigation suggests that similar mineral compositions in ancient microbialites could also be a marker of oxygenic photosynthesis, which is a major finding with respect to the interpretation of ancient microbial metabolisms.

## Conclusions

This study shows the important role of various microbial metabolisms in modifying their microenvironment by producing and degrading EOM in extant GSL microbial mats. The chemical changes induced by microbial activity in turn control the nucleation, structure and composition of the minerals produced in the mats. The mineralisation processes documented in the present study were likely active in numerous paleolakes. Future work will focus on the diagenetic transformations of mineral products, especially microbial Mg-Si phases, and further enhance the interpretations of ancient microbialites.

## Materials and Methods

### Geological mapping

Geological mapping was performed during three field campaigns in June 2013, September 2013 and November 2015, on the northwestern shore of Antelope Island, extending from White Rock Bay up to Red Rocks Canyon (Supplementary Fig. S1). Field observations were combined with aerial and satellite images. Aerial images were obtained from Google Earth Pro 7.1.2 (from State of Utah, USDA Farm Service Agency and NASA; unknown remote-sensors), the Utah Automated. Geographic Reference Center (2014 NAIP 1 Meter 4-bands (RGB and infrared) Orthophotography, used in 3-bands RGB natural colour) and the USGS EarthExplorer (aerial photo single frames, NAPP, NAHP; mixing black and white, RGB and infrared bands). Satellite images correspond to Digitalglobe® images (WV02 spacecraft, Imaging bands Pan-MS1-MS2; obtained from Garmin Birdseye) and Landsat images (NASA Landsat Program, 1972 to 2015, L1-5 MSS/L4-5 TM/L7 ETM+ SLC-On/L7 ETM+ SLCOff/L8 OLI/TIRS, Sioux Falls, USGS, 08/07/72-10/13/2015). Aerial images have been imported in ArcGIS in order to perform the mapping. The first approach consists in converting the pixel showing microbial deposits into black pixel and integrated them into a shapefile layer. The percentage of points (i.e. microbial deposits) per surface unit was used in order to calculate the density of the microbial structures (in ArcGIS; Point density tool, 15 m output cells and 50 m circular neighbourhood). Calculations were difficult below a pixel resolution of 15 m due to the high amount of point; thereby it was completed with a visual approach improving the mapping resolution. For this we defined high and low densities areas of microbial deposits in aerial images which match with the perception in the field. These results have been emplaced in a metric topographic map for the GSL floor. This map was constructed by extrapolating the feet-unit map provided by Baskin and Allen^23^.

### Microelectrode and biogeochemical measurements

Samples were collected between Bridger Bay and Red Rocks Canyon along the shoreline of Antelope Island, but also offshore Eardley Spit (Supplementary Fig. S1). Part of the lithifying mats samples were stored at 4 °C directly after being collected; in order to preserve EOM for Confocal Laser Scanning Microscopy (CLSM) and Cryo-SEM analyses, other subsamples were fixed in filtered (0.22 μm) lake water with the addition of formaldehyde (2% final concentration). Dissolved oxygen concentration profiles (vertical resolution = 250 μm; Fig. 1A) were measured *ex situ* in lithifying microbial mats under natural light, within 2 hours of sampling. The measurements were taken using polarographic needle microelectrodes in combination with a picoammeter (Unisense, Aarhus, Denmark^50^). Selected mat samples were transferred to the lab, where they were submersed in *ca*. 5 cm of GSL site water, and incubated in a greenhouse for 2 weeks. Subsequently, microelectrode profiles of pH, HS^−^ and O_2_ profiles were measured using the Unisense Field Microsensor Multimeter system. (Fig. 1C).

The sulfate reduction activity was mapped in two mat samples using 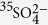 coated silver foil^32^. During a 4-hr incubation of vertically cut mat sample, sulfate reduction produced sulfide, which precipitated as Ag_2_^35^S onto the foil surface. The distribution of the Ag_2_^35^S radioactivity was mapped using a BioRad Molecular Imager System GS-525 (Hercules, California) radioactive gel scanner. This method accurately documents the metabolic activity of sulfate reducers as shown by comparison of Ag-foil maps and confocal scanning laser microscopy using SRB-specific dsrAB probes and reflects traditional sulfate reduction measurements in which ^35^SO_4_^2−^ is injected in sediment cores^32^,^51^. Pixel maps of sulfate-reducing activity were generated using Adobe software. The colour of the pixels is indicative of the rate of reduction (i.e., red pixels represent high, orange intermediate and yellow pixels lower activity; Fig. 1B).

### Optical and Scanning Electron Microscopy

A slab and thin section were prepared from each microbial mat and microbialite sample (Supplementary Table S1). Thin sections were examined first using polarizing light microscopy (Eclipse Ci-Pol Nikon), and then with a FEI Quanta 250 environmental scanning electron microscope, under low vacuum. X-ray spectral microanalyses and X-ray maps were performed using energy dispersive spectrometry (EDS; EDAX Apollo XL; I2 M, Bordeaux, France). Additional X-ray spectral microanalyses were performed on a Jeol 5600 LV SEM equipped with an EDS analyser (silicon drift detector BRUKER). The analytical conditions were an acceleration voltage of 15 kV, probe current at 1 nA, working distance at 16.5 mm, and counting time is 60 s. The standards used for EDS consist of albite (Na, Al, Si), almandine (Mg, Fe), diopside (Ca), orthoclase (K), spessartine (Mn) and Ti metal (Ti).

In order to observe the three-dimensional relationships between the EOM and the minerals, one unimpregnated mat sample (WP136A-2) was viewed at high magnification by combining cryofixation and SEM (Phillips XL 30, field emission gun in University of Connecticut, Groton, USA).

### Confocal Laser Scanning Microscopy and Raman Spectrometry

Samples preparation and analyses were carried out as described by Gérard^52^ without modifications. The thin sections of the sample WP20A-1 were observed under Confocal Laser Scanning Microscopy (CLSM), at the Institut de Physique du Globe de Paris (France), using a FluoView FV1000 CLSM with a spectral resolution of 2 nm and a spatial resolution of 0.2 mm (Olympus, Tokyo, Japan) coupled to an Invia Raman Spectrometer (Renishaw, Wotton-under-Edge, UK)^52^. Fluorescence image stacks were obtained by using 405-nm laser diode, 488-nm multiline argon and 543-nm helium-neon-green lasers (at 5%, 10% and 30% of maximum power, respectively). A 514-nm Ar laser source was used for Raman spectral analyses.

### Mineralogy

The organic matter was removed in all samples by reaction with H_2_O_2_ (30%). X-ray diffractometry (Siemens D500 Bragg-Brentano equipped with scintillation detector, using 30 kV Cu-Kα radiation) was successively performed on the bulk, sieved (fraction <100 μm) and clay fraction (<2 μm) of the samples. The clay fraction was analysed on oriented and unoriented powders. All mineral phases were determined using Bruker AXS software Eva (Diffract +14.0).

Micro-FTIR (Fourier Transform Infrared spectroscopy) was performed on the same fractions as XRD analyses. 1 mg of the powder was mixed with 150 mg of potassium bromide. The powders were pressed for 5 minutes under 8 tons, then for 1 minute under 10 tons and finally put in the oven for 3 hours (successively at 150, 200, 300 °C). Then powders were then analysed with a Fourier Nicolet spectrometer (detector: DTGS CSI, separating: CSI) in the 300–4000 cm^−1^ wavenumber range (spectral resolution of 0.125 cm^−1^; intervals of 4 cm^−1^), at the HYDRASA laboratory (Poitiers, France).

### Inorganic geochemistry

In order to avoid composite signals, stable isotopes (δ^18^O and δ^13^C) were measured on almost pure carbonate mineral phases (checked with XRD analyses). Different type of microbial and non-microbial minerals were selected (Fig. S7). Individual ooids and pellets were selected under a dissecting microscope. After organic matter removal, lithifying mats mineral fractions were ground in planetary ball mill Retsch PM100 during 20 min and then sieved at <100 μm to remove trapped grain (ooids mainly). For microbialites, unimpregnated and polished slabs were used. The white aragonite patches were first micro-drilled (Proxxon MF70 drill; 0.3 mm drill). Then, the periphery of patches, made of a mix of dolomite and aragonite were drilled. Subsequently, the powder was then soaked in acetic acid (1 mol L^−1^) for 4 h in order to dissolve aragonite and preserve dolomite. Consequently, dolomite and aragonite were analysed separately.

Stable isotope analyses were performed at the Pole Spectrométrie Océan in Brest (France). The samples were first ground in an agate mortar and sieved to ensure a grain size lower than 140 μm. CO_2_ was extracted by dissolution with 100% H_3_PO_4_ at 70 °C for 12 h in helium flushed Labco Exetainer® vials. Stable carbon and oxygen isotopic compositions of the evolved CO_2_ were measured using a gas chromatograph coupled to an isotope ratio mass spectrometer (Gas Bench 2 coupled to a Delta V Plus from Thermo Fisher), with helium as carrier gas. Three internal standards (CA21 – Rennes 0 – Across) were used to calibrate the δ^13^C_sample/ref_ data provided by the GC-IRMS relative to the V-PDB scale. These standards have been calibrated relative to V-PDB using two international standards, NBS19 and IAEACO1 (IAEA catalogue). Results are given in the usual δ-notation relative to the international Standards PDB for the δ^13^C and for the δ^18^O. The external reproducibility for δ^13^C and δ^18^O measurements is of 0.1‰ and 0.15‰, respectively (1σ). Each sample was measured twice and the average of two analyses is reported in the Supplementary Fig. S13.

### Dating

Radiocarbon ages (∆^14^C) were measured on two samples at the Atmosphere and Ocean Research Institute, the University of Tokyo (Japan) using Single Stage Accelerator Mass Spectrometry. Sample preparations and analytical conditions vary depending on the sample size and are described in detail^53^. Ages, calibrated using CalPal online (http://www.calpal-online.de/), are given in Supplementary Fig. S1.

### Water chemistry and geochemical modelling

Water chemical compositions come from the USGS water resources database (http://www.usgs.gov/water/). Samples were taken at two dates close to the sampling periods, 31 May 2013 (depth 0.2 m) and 27 July 2013 (depth 0.1 m) at the GSL 3510 station, which is located *ca*. 10 km west of the sampling sites (coordinates 40°.53′.56″N; 112°.20′.56″W). Data (Supplementary Figs S7 and S8) were entered in the geochemical modeling software Phreeqc v.3-A^54^ to determine the saturation index of the amorphous silica, dolomite and aragonite as a function of pH, using the Pitzer model^55^. These data were compared to the mineral composition of microbial mats and microbialites.

## Additional Information

**How to cite this article**: Pace, A. *et al*. Microbial and diagenetic steps leading to the mineralisation of Great Salt Lake microbialites. *Sci. Rep.* **6**, 31495; doi: 10.1038/srep31495 (2016).

## Supplementary Material

Supplementary Information

## References

[b1] Dupraz C. . Processes of carbonate precipitation in modern microbial mats. Earth-Sci. Rev. 96, 141–162 (2009).

[b2] Burne R. V. & Moore L. S. Microbialites: Organosedimentary Deposits of Benthic Microbial Communities. PALAIOS 2, 241–254 (1987).

[b3] Allwood A. C., Walter M. R., Kamber B. S., Marshall C. P. & Burch I. W. Stromatolite reef from the Early Archaean era of Australia. Nature (2006). 10.1038/nature04764 16760969

[b4] Riding R. Microbial carbonates: the geological record of calcified bacterial–algal mats and biofilms. Sedimentology 47, 179–214 (2000).

[b5] Loope G. R., Kump L. R. & Arthur M. A. Shallow water redox conditions from the Permian–Triassic boundary microbialite: The rare earth element and iodine geochemistry of carbonates from Turkey and South China. Chem. Geol. 351, 195–208 (2013).

[b6] Eigenbrode J. L. & Freeman K. H. Late Archean rise of aerobic microbial ecosystems. Proc. Natl. Acad. Sci. 103, 15759–15764 (2006). 17043234 10.1073/pnas.0607540103 PMC1635076

[b7] Kamber B. S. & Webb G. E. The geochemistry of late Archaean microbial carbonate: implications for ocean chemistry and continental erosion history. Geochim. Cosmochim. Acta 65, 2509–2525 (2001).

[b8] Anadón P., Canet C. & Friedrich W. L. Aragonite stromatolitic buildups from Santorini (Aegean Sea, Greece): Geochemical and palaeontological constraints of the caldera palaeoenvironment prior to the Minoan eruption (ca 3600 yr bp). Sedimentology 60, 1128–1155 (2013).

[b9] Arp G., Reimer A. & Reitner J. Microbialite Formation in Seawater of Increased Alkalinity, Satonda Crater Lake, Indonesia. J. Sediment. Res. 73, 105–127 (2003).

[b10] Des Marais D. J. Biogeochemistry of Hypersaline Microbial Mats Illustrates the Dynamics of Modern Microbial Ecosystems and the Early Evolution of the Biosphere. Biol. Bull. 204, 160–167 (2003). 12700147 10.2307/1543552

[b11] Warren J. K. Evaporites: Sediments, Resources and Hydrocarbons (Springer Science & Business Media, 2006).

[b12] Vasconcelos C. & McKenzie J. A. Microbial Mediation of Modern Dolomite Precipitation and Diagenesis Under Anoxic Conditions (Lagoa Vermelha, Rio de Janeiro, Brazil). J. Sediment. Res. 67, (1997).

[b13] Arp G. . Photosynthesis versus Exopolymer Degradation in the Formation of Microbialites on the Atoll of Kiritimati, Republic of Kiribati, Central Pacific. Geomicrobiol. J. 29, 29–65 (2012).

[b14] Dupraz C., Fowler A., Tobias C. & Visscher P. T. Stromatolitic knobs in Storr’s Lake (San Salvador, Bahamas): a model system for formation and alteration of laminae. Geobiology 11, 527–548 (2013). 24118887 10.1111/gbi.12063

[b15] Visscher P. T. . In Environmental Electrochemistry: Analyses of Trace Element Biogeochemistry (2002).

[b16] Decho A. W., Visscher P. T. & Reid R. P. Production and cycling of natural microbial exopolymers (EPS) within a marine stromatolite. Palaeogeogr. Palaeoclimatol. Palaeoecol. 219, 71–86 (2005).

[b17] Decho A. W. Microbial biofilms in intertidal systems: an overview. Cont. Shelf Res. 20, 1257–1273 (2000).

[b18] Braissant O. . Exopolymeric substances of sulfate-reducing bacteria: Interactions with calcium at alkaline pH and implication for formation of carbonate minerals. Geobiology 5, 401–411 (2007).

[b19] Burne R. V. . Stevensite in the modern thrombolites of Lake Clifton, Western Australia: A missing link in microbialite mineralization? Geology 42, 575–578 (2014).

[b20] Zeyen N. . Formation of low-T hydrated silicates in modern microbialites from Mexico and implications for microbial fossilization. Front. Earth Sci. 64, 10.3389/feart.2015.00064 (2015).

[b21] Tosca N. J., Macdonald F. A., Strauss J. V., Johnston D. T. & Knoll A. H. Sedimentary talc in Neoproterozoic carbonate successions. Earth Planet. Sci. Lett. 306, 11–22 (2011).

[b22] Bouton A. . Enhanced development of lacustrine microbialites on gravity flow deposits, Great Salt Lake, Utah, USA. Sediment. Geol. 341, 1–12 (2016).

[b23] Baskin R. L. & Allen D. V. Bathymetric map of the south part of Great Salt Lake (2005).

[b24] Rupke A. L. & McDonald A. Great Salt Lake Brine Chemistry Database 1966–2011, 1–11 (2012).

[b25] Chidsey T. C., Berg M. D. V. & Eby D. E. Petrography and characterization of microbial carbonates and associated facies from modern Great Salt Lake and Uinta Basin’s Eocene Green River Formation in Utah, USA. Geol. Soc. Lond. Spec. Publ. 418, SP418–SP940 (2015).

[b26] Lynch K. L. . Near-infrared spectroscopy of lacustrine sediments in the Great Salt Lake Desert: An analog study for Martian paleolake basins. J. Geophys. Res. Planets 120, 2014JE004707 (2015).

[b27] Eardley A. J. Sediments of Great Salt Lake, Utah. AAPG Bull. 22, 1305–1411 (1938).

[b28] Collister J. W. & Schamel S. In Great Salt Lake: An Overview of Change, Utah Department of Natural Resources Special Publication 127–142 (2002).

[b29] Wilson M. J. Clay Mineralogy: Spectroscopic and Chemical Determinative Methods (Springer Science & Business Media, 1994).

[b30] Gallagher K. L., Kading T. J., Braissant O., Dupraz C. & Visscher P. T. Inside the alkalinity engine: the role of electron donors in the organomineralization potential of sulfate-reducing bacteria. Geobiology 10, 518–530 (2012). 22925453 10.1111/j.1472-4669.2012.00342.x

[b31] Lippmann F. Sedimentary carbonate minerals: minerals, rocks and inorganic materials (Springer-Verlag Berlin, 1973).

[b32] Visscher P. T., Reid R. P. & Bebout B. M. Microscale observations of sulfate reduction: Correlation of microbial activity with lithified micritic laminae in modern marine stromatolites. Geology 28, 919–922 (2000).

[b33] Land L. S. Failure to Precipitate Dolomite at 25 °C fromDilute Solution Despite 1000-Fold Oversaturation after32 Years. Aquat. Geochem. 4, 361–368 (1998).

[b34] Van Lith Y., Warthmann R., Vasconcelos C. & Mckenzie J. A. Sulphate-reducing bacteria induce low-temperature Ca-dolomite and high Mg-calcite formation. Geobiology 1, 71–79 (2003).

[b35] Krause S. . Microbial nucleation of Mg-rich dolomite in exopolymeric substances under anoxic modern seawater salinity: New insight into an old enigma. Geology 43, 906–906 (2015).

[b36] Roberts J. A. . Surface chemistry allows for abiotic precipitation of dolomite at low temperature. Proc. Natl. Acad. Sci. 110, 14540–14545 (2013). 23964124 10.1073/pnas.1305403110 PMC3767548

[b37] Swart P. K. The geochemistry of carbonate diagenesis: The past, present and future. Sedimentology 62, 1233–1304 (2015).

[b38] Andres M. S., Sumner D. Y., Reid R. P. & Swart P. K. Isotopic fingerprints of microbial respiration in aragonite from Bahamian stromatolites. Geology 34, 973–976 (2006).

[b39] Souza-Egipsy V., Wierzchos J., Ascaso C. & Nealson K. H. Mg–silica precipitation in fossilization mechanisms of sand tufa endolithic microbial community, Mono Lake (California). Chem. Geol. 217, 77–87 (2005).

[b40] Bontognali T. R. R. . Smectite synthesis at low temperature and neutral pH in the presence of succinic acid. Appl. Clay Sci. 101, 553–557 (2014).

[b41] Myshrall K. L. . Biogeochemical cycling and microbial diversity in the thrombolitic microbialites of Highborne Cay, Bahamas. Geobiology 8, 337–354 (2010). 20491947 10.1111/j.1472-4669.2010.00245.x

[b42] Reid R. P. . The role of microbes in accretion, lamination and early lithification of modern marine stromatolites. Nature 406, 989–992 (2000). 10984051 10.1038/35023158

[b43] Couradeau E. . Cyanobacterial calcification in modern microbialites at the submicrometer scale. Biogeosciences 10, 5255–5266 (2013).

[b44] Kaźmierczak J. . Hydrochemistry and microbialites of the alkaline crater lake Alchichica, Mexico. Facies 57, 543–570 (2011).

[b45] Awramik S. M. & Buchheim H. P. Giant stromatolites of the Eocene Green River Formation (Colorado, USA). Geology 43, 691–694 (2015).

[b46] Sarg J. F., Suriamin N., TÌnavsuu-Milkeviciene K. & Humphrey J. D. Lithofacies, stable isotopic composition, and stratigraphic evolution of microbial and associated carbonates, Green River Formation (Eocene), Piceance Basin, Colorado. AAPG Bull. 97, 1937–1966 (2013).

[b47] Tettenhorst R. & Moore G. E. Jr. Stevensite Oolites from the Green River Formation of Central Utah. J. Sediment. Res. 48, 587–594 (1978).

[b48] Wasson M. S., Saller A., Andres M., Self D. & Lomando A. Lacustrine microbial carbonate facies in core from the lower Cretaceous Toca Formation, Block 0, offshore Angola. Am. Assoc. Pet. Geol. Hedberg Conf. ‘Microbial Carbonate Reserv. Charact. Houst. TX 4–8 (2012).

[b49] Guven N. & Carney L. L. The hydrothermal transformation of sepiolite to stevensite and the effect of added chlorides and hydroxides. Clays Clay Miner. 27, 253–260 (1979).

[b50] Visscher. . Environmental Electrochemistry: Analyses of Trace Element Biogeochemistry (Oxford University Press, 2002).

[b51] Decho A. W. . Autoinducers extracted from microbial mats reveal a surprising diversity of N-acylhomoserine lactones (AHLs) and abundance changes that may relate to diel pH. Environ. Microbiol. 11, 409–420 (2009). 19196272 10.1111/j.1462-2920.2008.01780.x

[b52] Gérard E. . Specific carbonate–microbe interactions in the modern microbialites of Lake Alchichica (Mexico). ISME J. 7, 1997–2009 (2013). 23804151 10.1038/ismej.2013.81 PMC3965311

[b53] Yokoyama Y., Koizumi M., Matsuzaki H., Miyairi Y. & Ohkouchi N. Developing ultra small-scale radiocarbon sample measurement at the University of Tokyo. Radiocarbon 52, 310 (2010).

[b54] Parkhurst D. L. & Appelo C. A. J. Description of input and examples for PHREEQC version 3: a computer program for speciation, batch-reaction, one-dimensional transport, and inverse geochemical calculations. 519 (U.S. Geological Survey, 2013).

[b55] Pitzer K. S. Theory: Ion Interaction Approach, Chapter 7 in Activity Coefficients in Electrolyte Solutions Pytkowicz R. M. Ed. (CRC Press, Boca Raton, FL, 1979).

