# Supplementary material for: Microbial and diagenetic steps leading to the mineralisation of Great Salt Lake microbialites
Source: Sci Rep. 2016 Aug 16;6:31495. doi: 10.1038/srep31495 (PMC4985759; doi:10.1038/srep31495)
Supplement: Supplementary Information [file srep31495-s1.pdf]

Supplementary Information

Microbial and diagenetic steps leading to the mineralisation of Great Salt Lake microbialites

Aurélie Pace, Raphaël Bourillot, Anthony Bouton, Emmanuelle Vennin, Serge Galaup, Irina Bundeleva, Patricia Patrier, Christophe Dupraz, Christophe Thomazo, Pierre Sansjofre, Yusuke Yokoyama, Michel Franceschi, Yannick Anguy, Léa Pigot, Aurélien Virgone and Pieter T. Visscher

Supplementary Figures

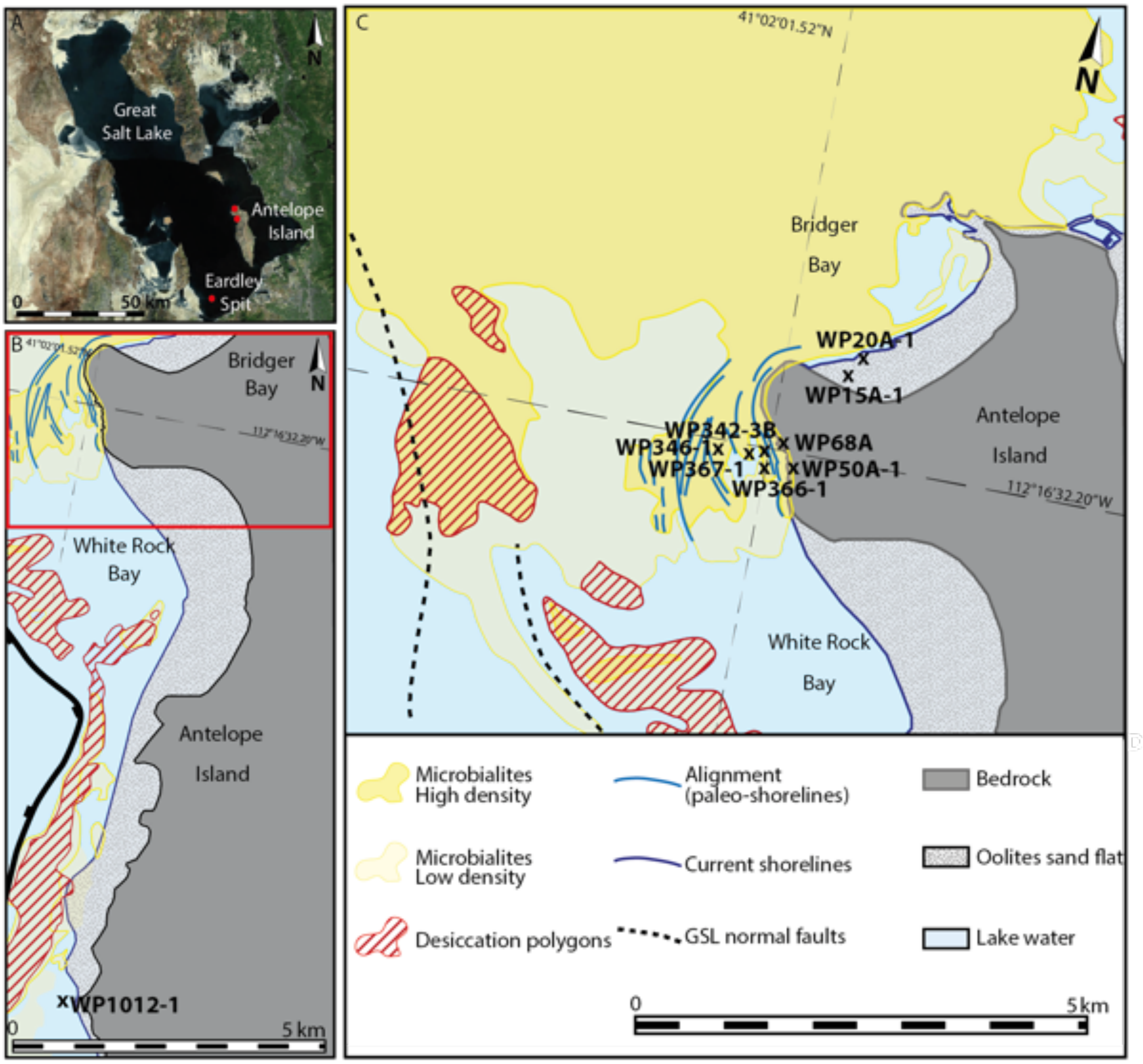

Supplementary Figure S1 (previous page): A. Satellite Image of Great Salt Lake (Google Earth; v.7.1.2.2041) with the two main sites of study. B and C. Sediment maps of the western margin of Antelope Island, indicating microbialite distribution (GSL faults from AGCR Database) and the location of sampling sites. The maps were generated with field observations combined with aerial and satellite images. Aerial images used for mapping were obtained from Google Earth Pro 7.1.2 (from State of Utah, USDA Farm Service Agency and NASA; unknown remote-sensors), the Utah Automated Geographic Reference Center (http://gis.utah.gov) and the USGS EarthExplorer (http://earthexplorer.usgs.gov). Satellite images used for mapping correspond to Digitalglobe images (https://www.digitalglobe.com) and Landsat images (NASA Landsat Program, 1972 to 2015, L1-5 MSS/L4-5 TM/L7 ETM+ SLC-On/L7 ETM+ SLCOff/L8 OLI/TIRS, Sioux Falls, USGS, 08/07/72-10/13/2015). All images have been imported in ArcGIS 10.2.1 in order to perform the mapping.

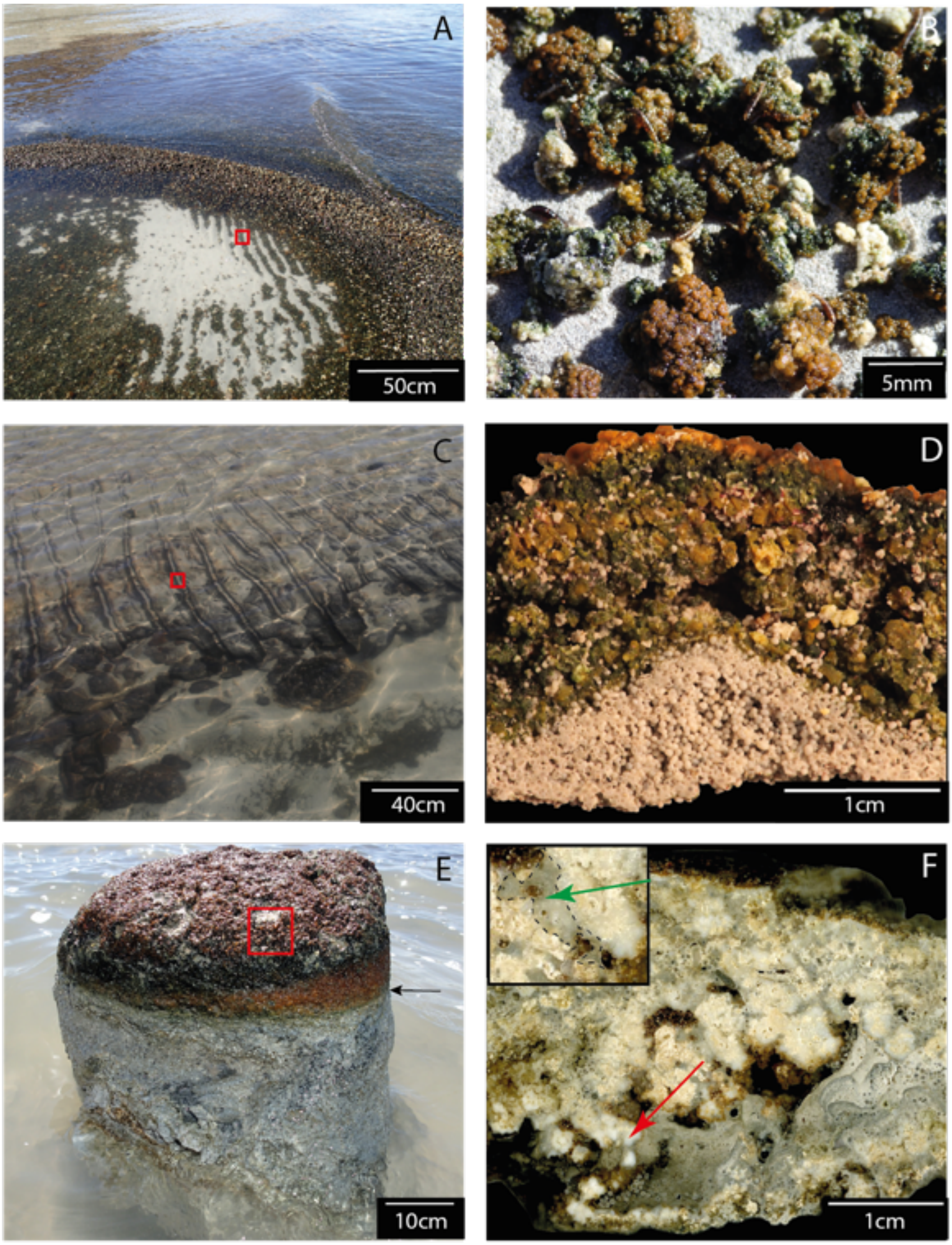
A
50cm
B
5mm
C
40cm
D
1cm
E
10cm
F
1cm

Supplementary Figure S2 (previous page): Photographs (field overview and detailed hand samples) of microbial mats and microbialites. A. Reworked fragments of lithifying microbial mats. B. Detail of A; C. Lithifying microbial mat developing on ooid sand ripple. D. Cross-section of the lithifying mat (C). E. Microbialite column exhumed from ooid sand. The black arrow indicates the boundary between the buried and exposed part. F. Polished slab of the microbial crust at the top of the column (E) showing white carbonate patches (red arrow: aragonite; green arrow: dolomite) forming an intestine-like, clotted fabric (thrombolite-type). The base of the crust has a laminated fabric (stromatolite-type).

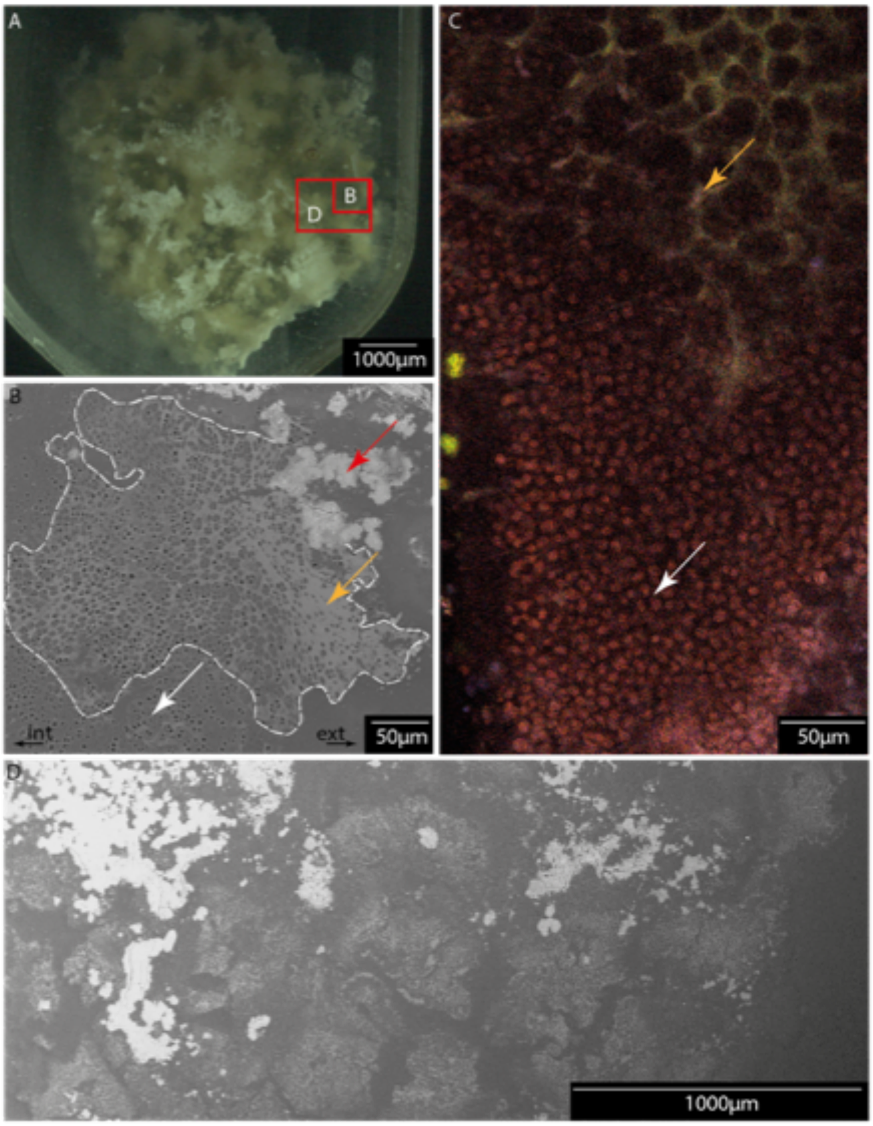

Supplementary Figure S3: SEM and CLSM observations inside a reworked fragment of a lithifying mat (sample WP20A-1). A. Scanned slab with location of B, C and D (red rectangle). B. SEM image of a coccoid cluster showing the spatial organization of mineralised and unmineralised zones: at the bottom and to the left, the EOM are not mineralised (white arrow). The white dotted line delineates a zone impregnated by Mg-Si phase (orange arrow). To the right, aragonite infills coccoids and covers EOM; int=interior of the sample; ext=exterior. C. CLSM image (same zone as Fig. 3A) of a similar zone in the same sample. The increase in yellowish fluorescence (reflecting Mg-binding) towards the top indicates a progressive impregnation of EOM by Mg-Si phase (orange arrow) within a photosynthetically active coccoid clusters (white arrow; red fluorescence). D. SEM image of a larger area, showing the clustered distribution of Mg-Si phases and aragonite, which is controlled by the organisation of the coccoid clusters.

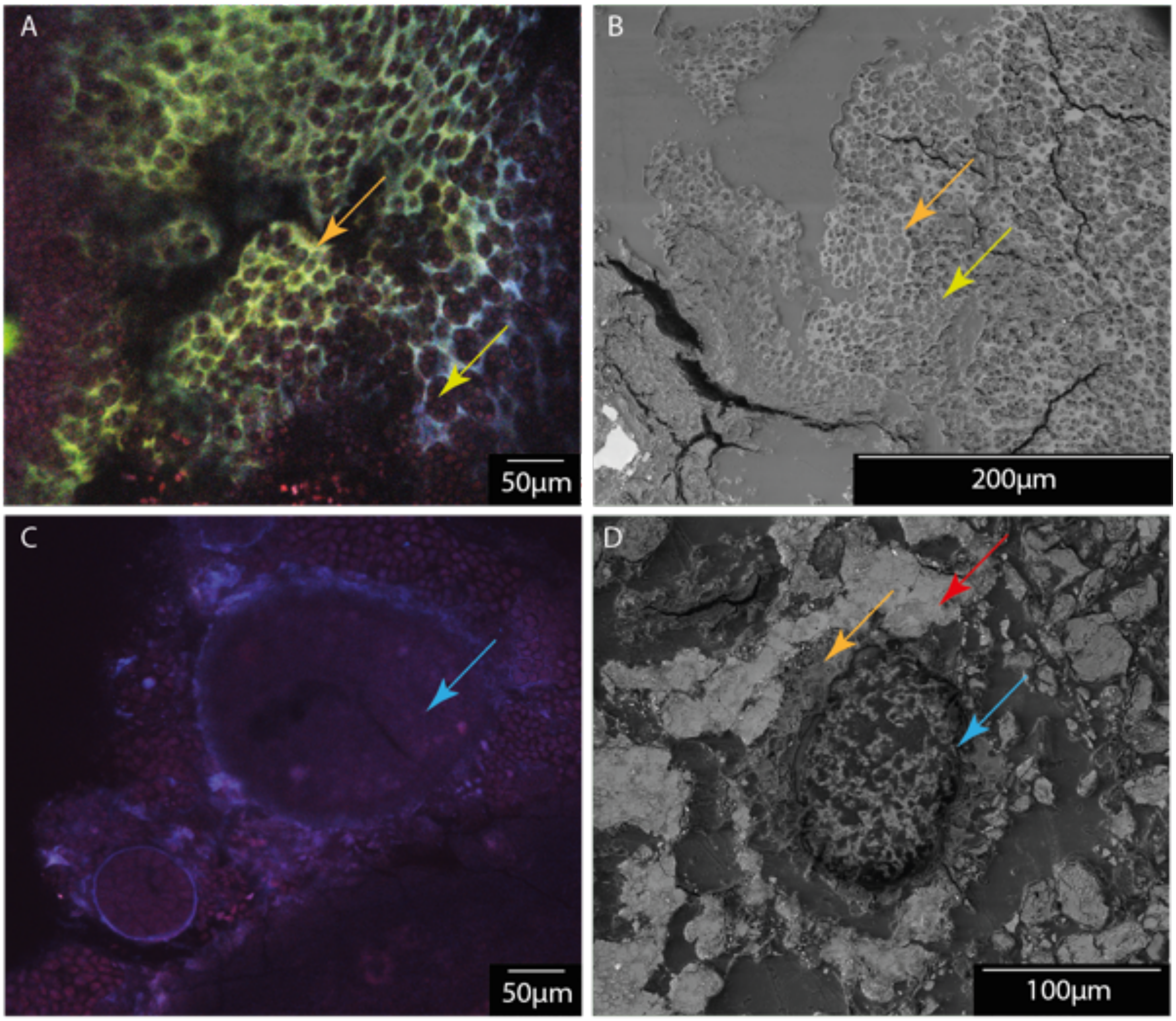

Supplementary Figure S4: Microscopic images of the two dominant coccoid cyanobacteria morphotypes. A and B are respectively CLSM and SEM images of a lithifying mat (WP20A-1). A and B. Coccoids of the first morphotype (type 1) are organized in small 1 to 8 cells colonies (yellow arrow) grouped in large 50 to 500 µm clusters. The Mg-Si phase nucleates on the EOM between type 1 coccoid colonies (orange arrow). C and D are respectively CLSM and SEM images of a lithifying mat (WP137A-1 and WP367-1). C. Coccoids of the second morphotype (type 2) forms tens to hundreds of cells colonies surrounded by an external sheath (blue arrow). D. A type 2 colony is surrounded by the Mg-Si phase covering the EOM (orange arrow) and aragonite patches (red arrow) infilling type 1 cells.

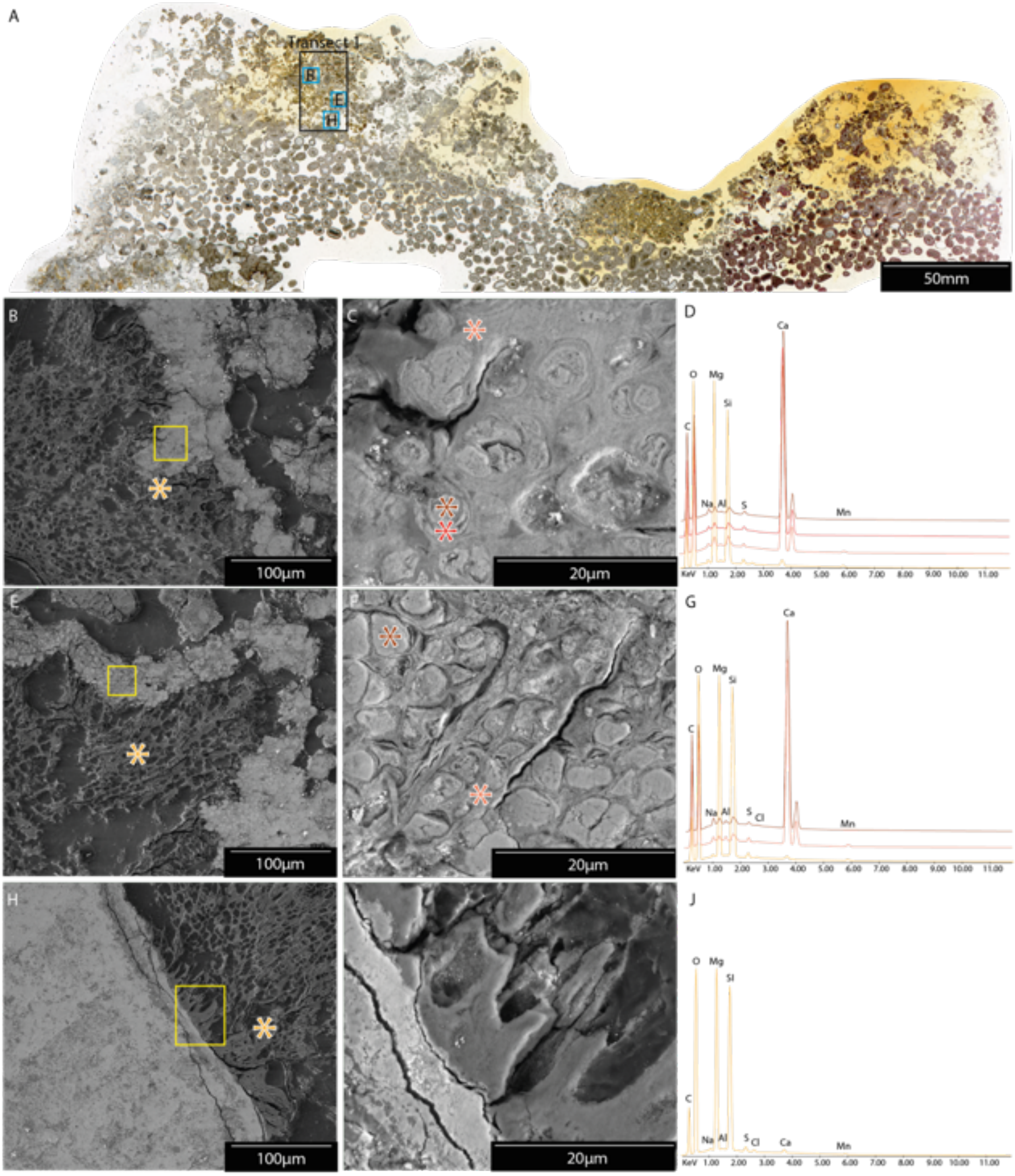
A
Transect 1
B
E
H
50mm
B
100µm
C
20µm
D
C
O
Mg
Si
Na
Al
S
Ca
Mn
KeV 1.00 2.00 3.00 4.00 5.00 6.00 7.00 8.00 9.00 10.00 11.00
E
100µm
20µm
G
C
O
Mg
Si
Na
Al
S
Cl
Ca
Mn
KeV 1.00 2.00 3.00 4.00 5.00 6.00 7.00 8.00 9.00 10.00 11.00
H
100µm
20µm
J
O
Mg
Si
C
Na
Al
S
Cl
Ca
Mn
KeV 1.00 2.00 3.00 4.00 5.00 6.00 7.00 8.00 9.00 10.00 11.00

Supplementary Figure S5 (previous page): SEM observations along a vertical profile in a lithifying mat (sample WP367-1). The microbial activity has been measured in the same sample (Fig. 1). A. Scanned thin section with location of the SEM images (blue rectangles). B, E and H are SEM pictures taken respectively from the top to the bottom of the mat. C, F and I correspond respectively to the zoom of B, E and H (yellow rectangle). D, G and J are EDS spectra measured respectively on B-C, E-F and H-I. Each spectrum has a colour, and the location of the corresponding analysis spot is indicated on images by an asterisk of the same colour. Brown spectra have been measured inside coccoid cell, red spectra have been measured in the wall of coccoid cell, coral spectra have been measured between coccoid cells and orange spectra have been measured in the EOM outside aragonite patches. In the aragonite patches, there is an increase in Mg and Si content from the inside of the cells, which are infilled with almost pure aragonite, towards the former EOM, made of a mix of aragonite and Mg-Si phase.

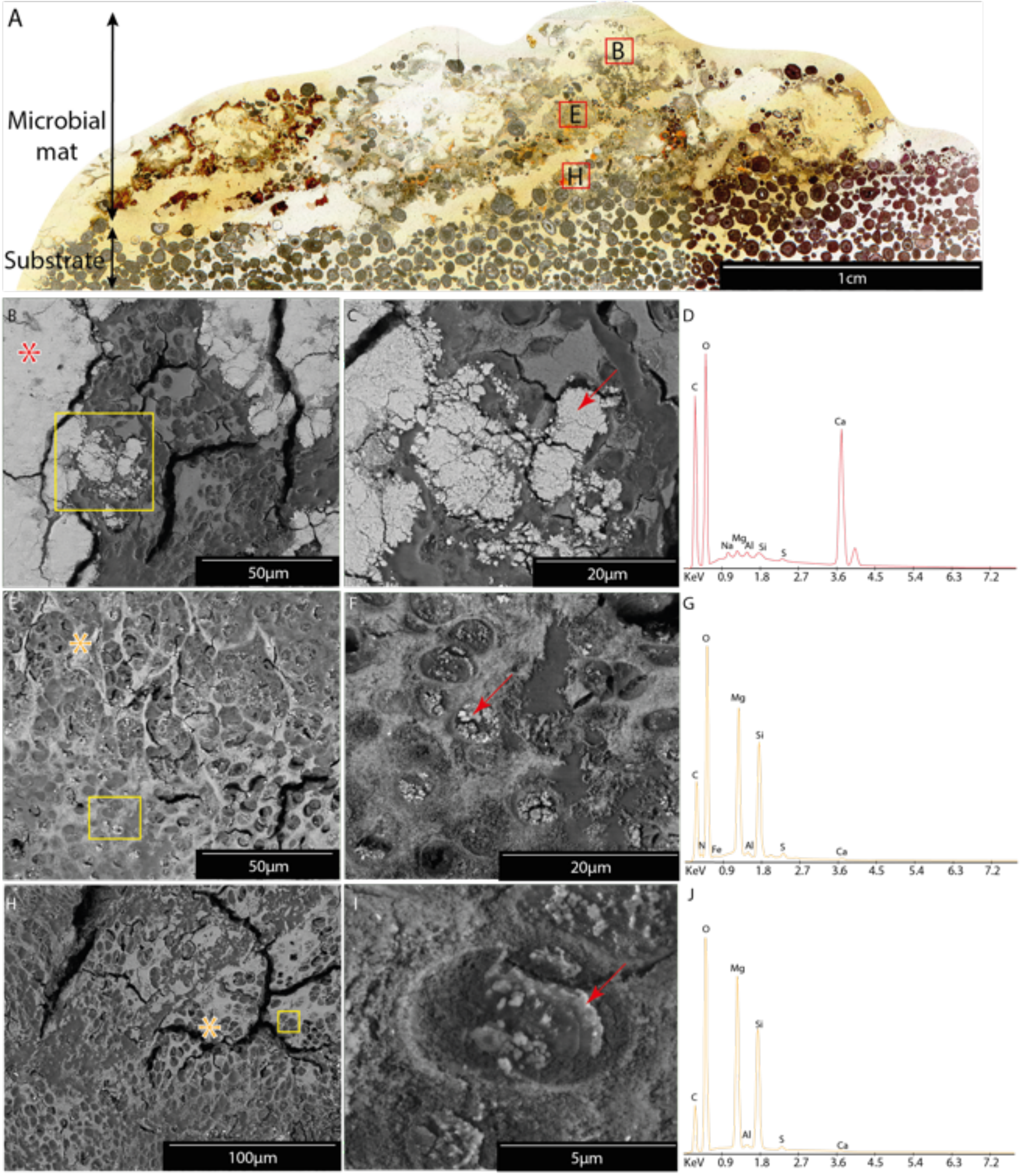
A
Microbial mat
Substrate
B
E
H
1cm
B
50µm
C
20µm
D
C
O
Ca
Na
Mg
Al
Si
S
KeV 0.9 1.8 2.7 3.6 4.5 5.4 6.3 7.2
E
50µm
F
20µm
G
O
Mg
Si
C
N
Fe
Al
S
Ca
KeV 0.9 1.8 2.7 3.6 4.5 5.4 6.3 7.2
H
100µm
I
5µm
J
O
Mg
Si
C
Al
S
Ca
KeV 0.9 1.8 2.7 3.6 4.5 5.4 6.3 7.2

Supplementary Figure S6 (previous page): SEM observations along a vertical profile in a lithifying mat (sample WP366-1). A. Scanned thin section with location of the SEM images (blue rectangles). B, E and H are SEM pictures taken respectively from the top to the bottom of the mat. C, F and I correspond respectively to the zoom of B, E and H (yellow rectangle). D, G and J are EDS spectra measured respectively on B-C, E-F and H-I. Each spectrum has a colour, and the location of the corresponding analysis spot is indicated on images by an asterisk of the same colour. The red spectrum has been measured in an aragonite patch, orange spectra have been measured in the EOM outside aragonite patches. This figure shows the nucleation of aragonite (red arrows) as rounded microcrystals inside cells.

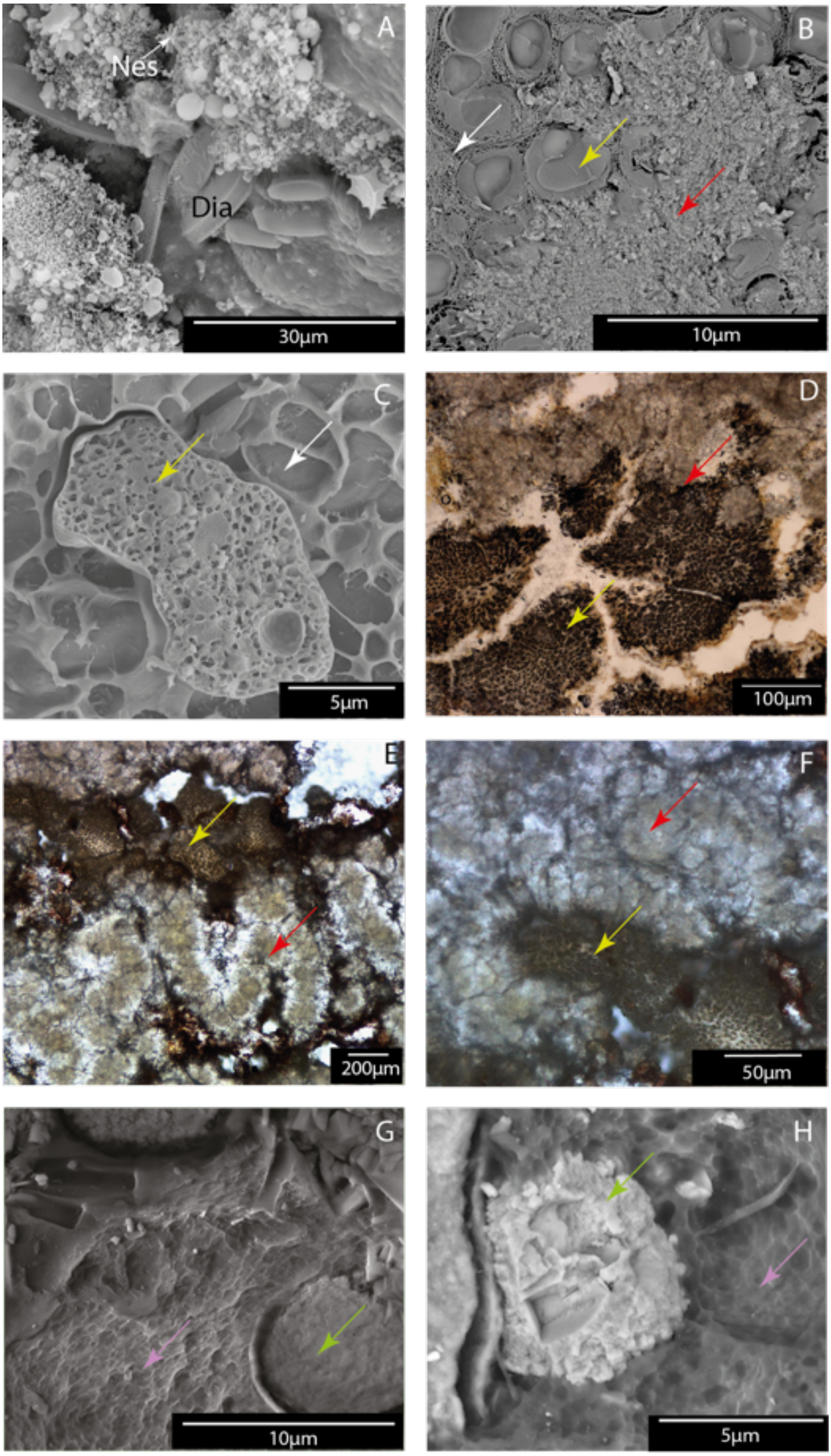
A
Nes
Dia
30µm
B
10µm
C
5µm
D
100µm
E
200µm
F
50µm
G
10µm
H
5µm

Supplementary Figure S7 (previous page): Microscopic observations of lithifying mats and microbialites. A - C are cryo-SEM images of a soft mat (WP136A-2); D - F depict polarizing microscope images of a lithified microbialite (WP342-3B); G and H show environmental SEM images of a lithified microbialite (WP342-3B). A. Diatoms (Dia) and authigenic minerals (including possible nesquehonite; Nes) at the surface a lithifying microbial mat. B. Progressive replacement of coccoid cells (yellow arrow) and EOM (white arrow) by aragonite (red arrow). C. Cluster of high density EOM encapsulating coccoid cyanobacteria (yellow arrow), surrounded by lower density EOM (white arrow). D. Clusters of coccoid cyanobacteria (yellow arrow) are progressively replaced by aragonite (red arrow). E. Aragonite acquires an intestine-like fabric (red arrow) on the coccoids cyanobacteria (yellow arrow) by replacing microbial clusters (yellow arrow). F. Detail of a cluster of coccoids (yellow arrow) progressively replaced by intestine-like aragonite (red arrow). G and H. Dolomite filling a spherical structure, possibly a former coccoid cyanobacteria (green arrow) in between EOM (purple arrow) and a patch of aragonite.

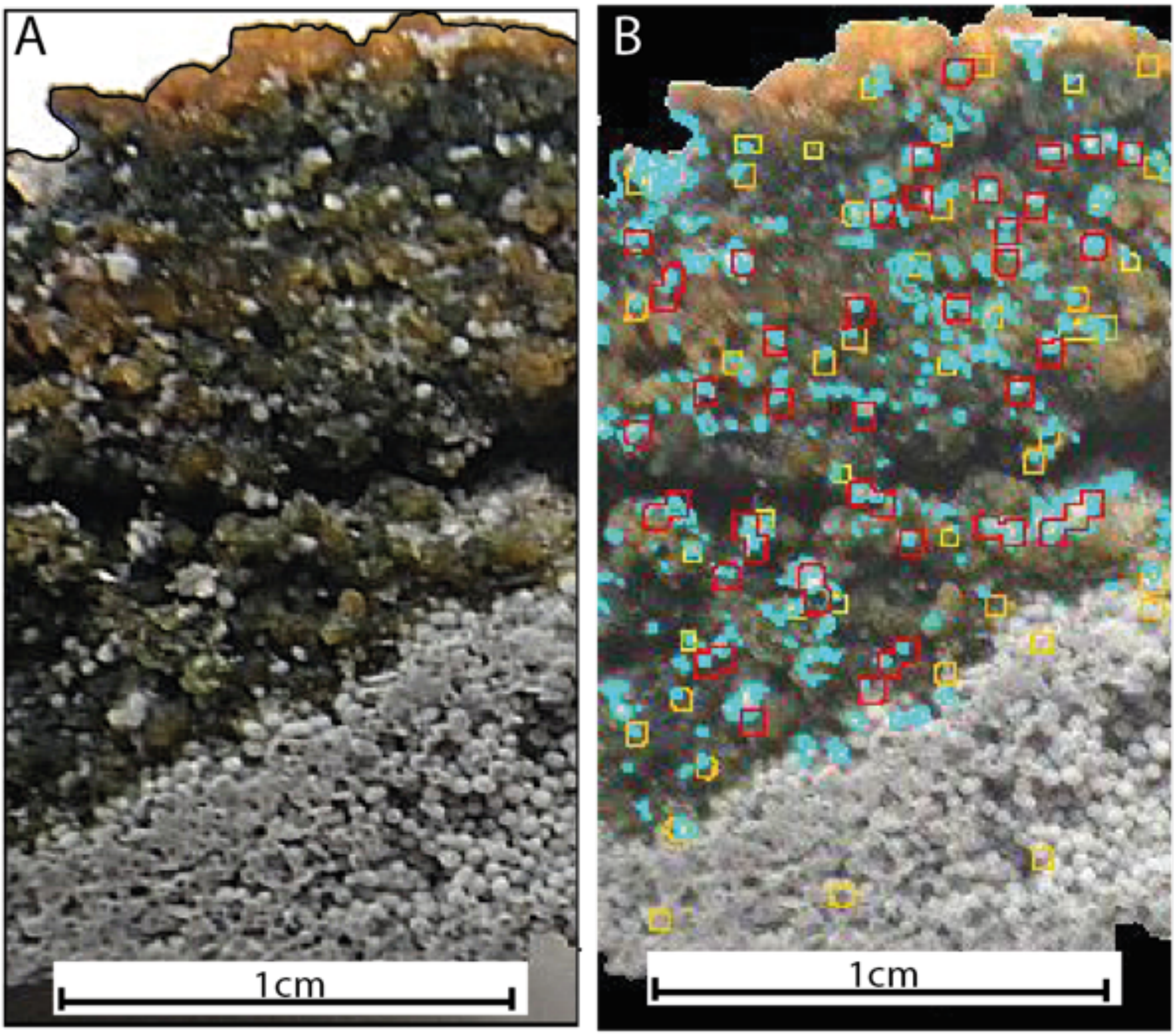

Supplementary Figure S8: Correspondence between sulfate reduction (SR) hotspots and aragonite patches in the lithifying mat (WP367-1; Fig. 1*B*). A. Lithifying mat. B. Map of SR activity by image processing (blue: aragonite, red: high SR activity, orange: medium SR activity, yellow: low SR activity).

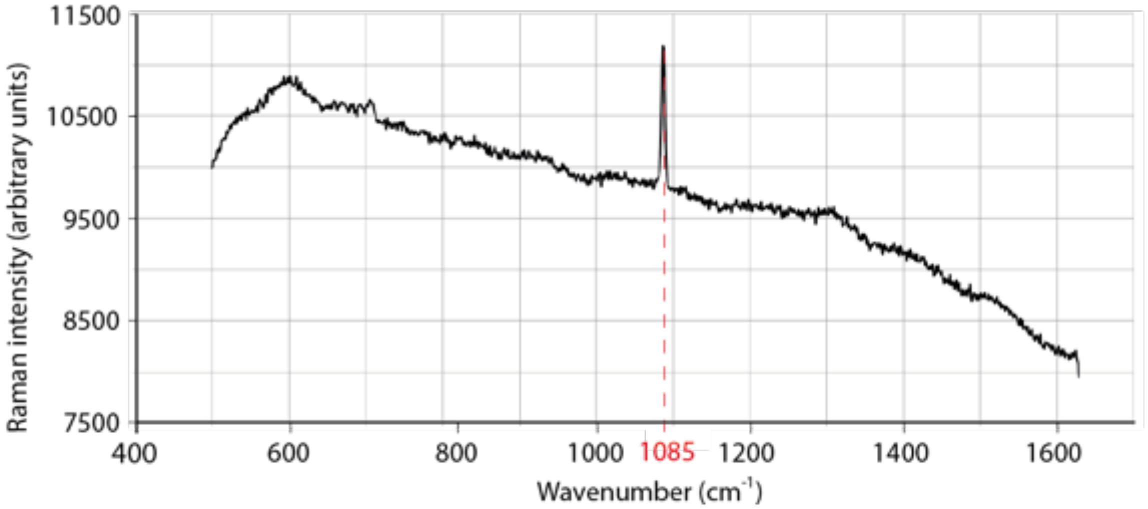

Supplementary Figure S9: Raman spectrum (for location see red arrow on Fig. 3F) showing a peak at 1085 cm^{-1} indicative of a $CaCO_3$ phase (here aragonite).

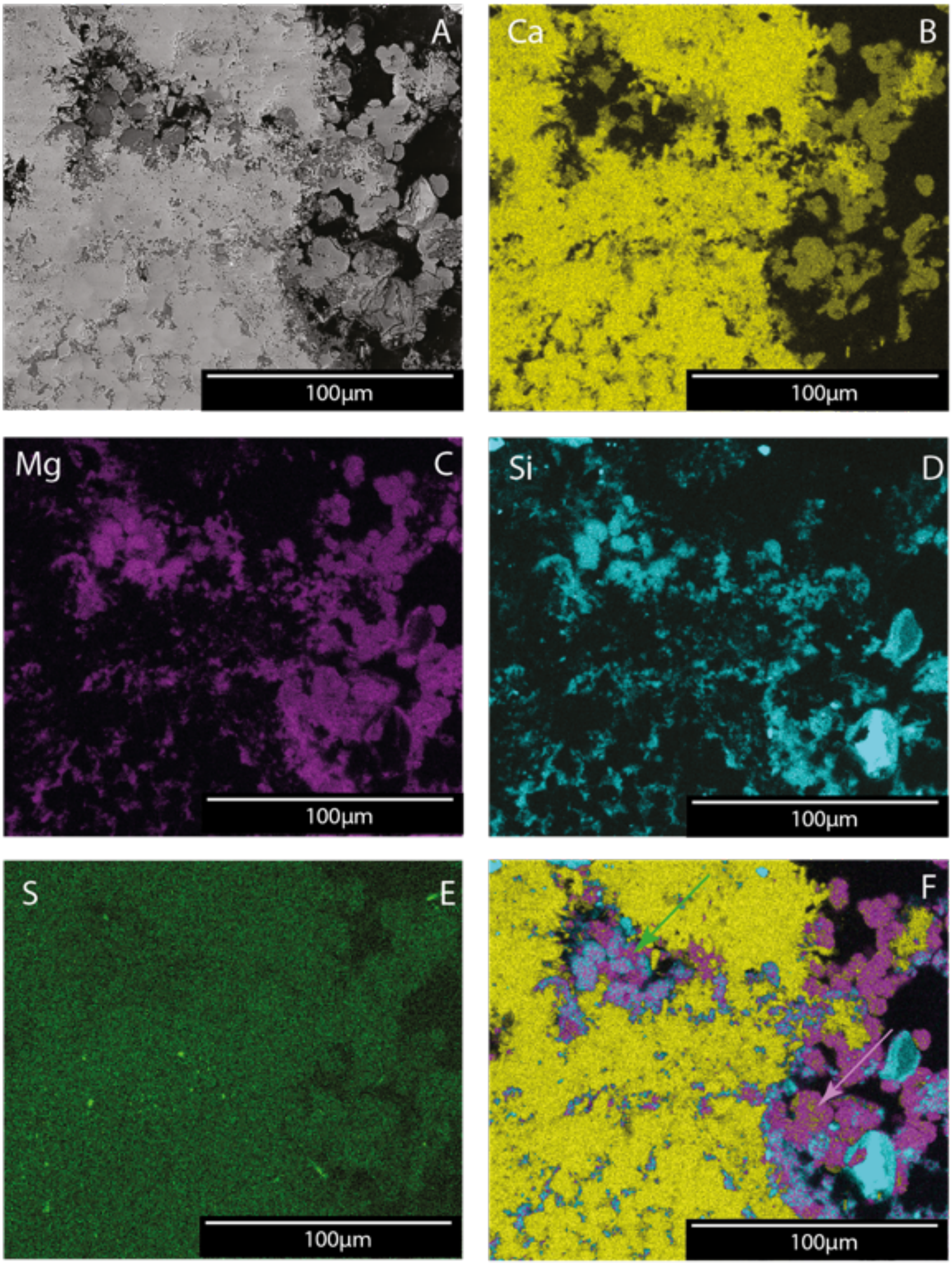

Supplementary Figure S10: Elemental EDS maps of a microbialite (WP342-3B). A. Back scattered electron image. B, C, D and E. respectively depict calcium, magnesium, silicon and sulfur maps. F. Overlay of Ca (yellow), Mg (purple) and Si (blue). Aragonite is yellow, dolomite is mixed purple and yellow (green arrow) and the Mg-Si phase is mixed purple and blue (purple arrow).

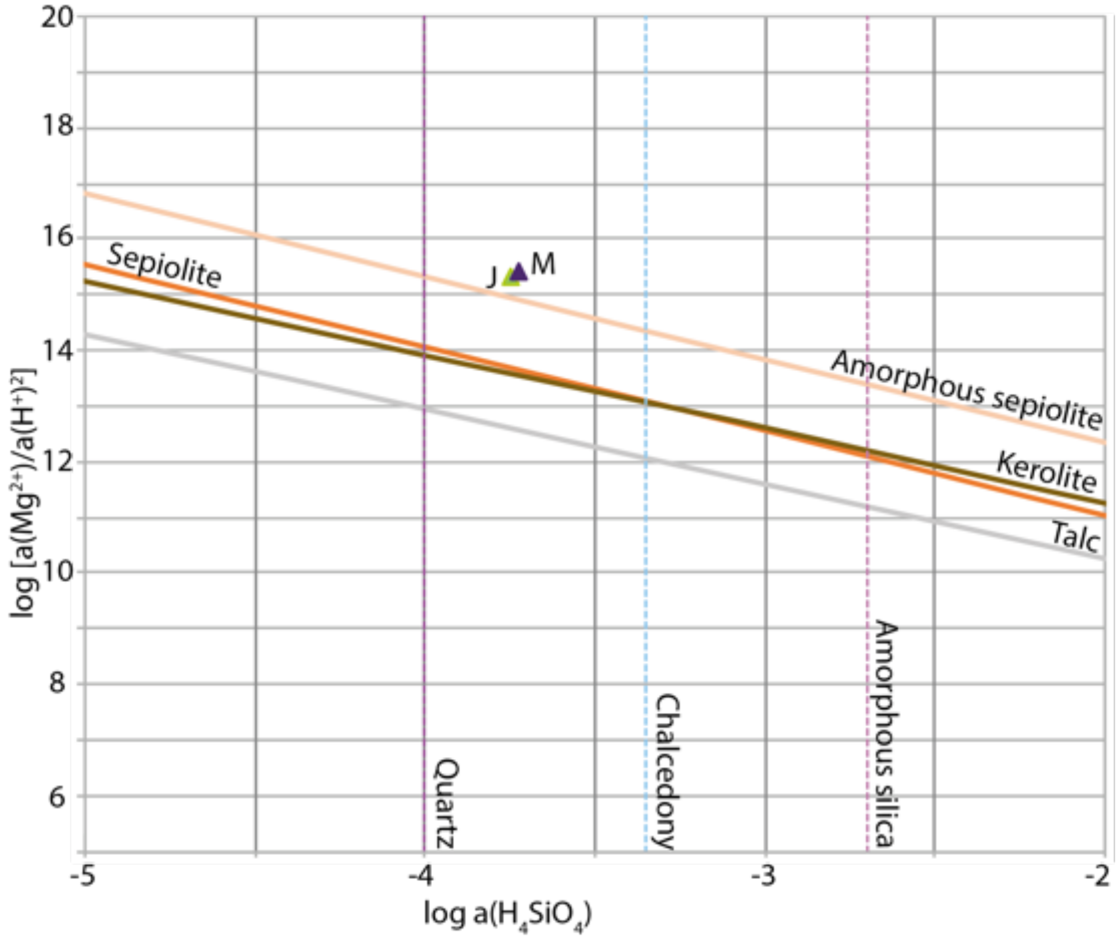

Supplementary Figure S11: Solubility diagram showing apparent solubilities of different Mg-silicate phases. Solubility lines of crystalline and amorphous phases are generated from the Pitzer model [1] on Phreeqc. Violet triangle represents the water solution sampled in May (T°= 13°C) and green triangle represents the water solution sampled in July (T°=26°C; see Supplementary Table 4).

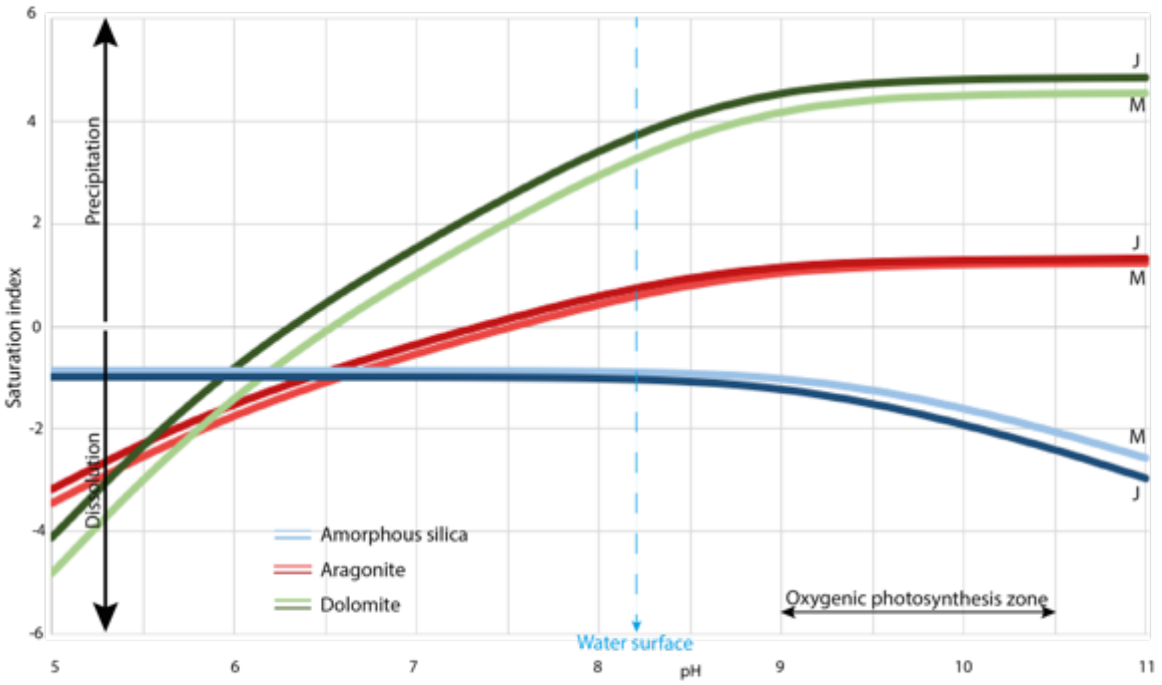

Supplementary Figure S12: Results of Phreeqc modelling based on GSL surface water composition (USGS; Supplementary Table S2). Saturation index as a function of the pH for three minerals (amorphous silica, aragonite and dolomite). Each mineral is represented by two curves showing the variation induced by seasonal temperature fluctuations (M: May; J: July).

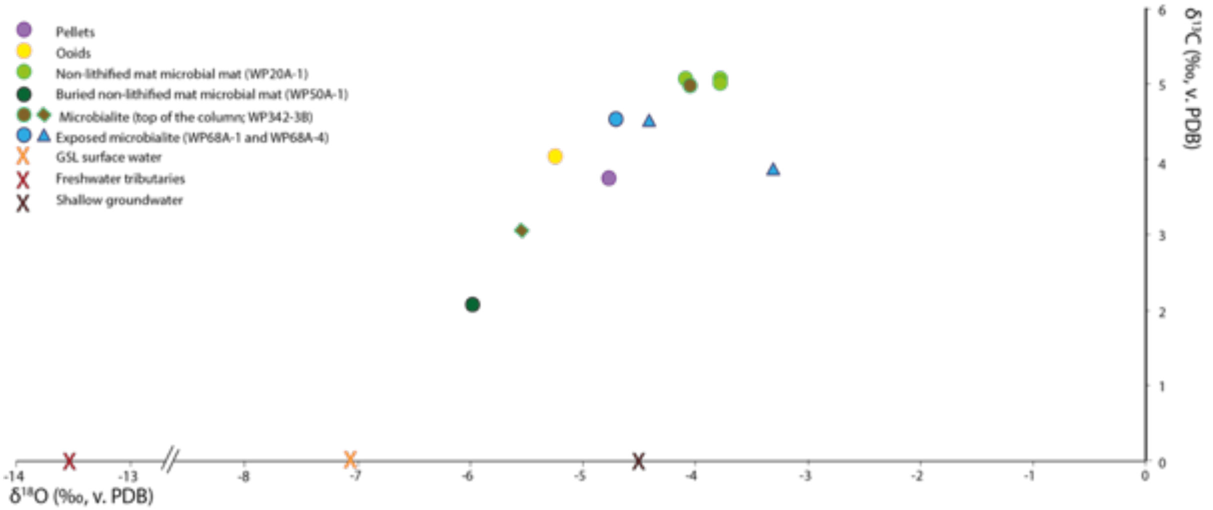

Supplementary Figure S13: Plot of $\delta^{18}O$ and $\delta^{13}C$ of mineral phases and GSL water. Circles: aragonite; Diamonds: dolomite; Triangles: calcite. $\delta^{18}O_{H2O}$ data from[2].

Supplementary Tables

Sample ID	Location	Depth (m)	Type	Morphology	Sub-sample type	Stable Isotopy (‰, v. PDB) $\delta^{13}C$	$\delta^{18}O$	$\Delta^{14}C$ Age (yrs BP. calibrated)
WP15A-1	N41°02.531'' W112°16.084''	0	Ooids		Bulk	4.03	-5.25	3517 ± 48
			Pellets		Bulk	3.76	-4.77	
WP20A-1	N41°02.567'' W112°16.088''	0	Lithifying microbial mat	Reworked fragments of microbial mats	Bulk	5.06	-4.09	
					Fraction < 250 μm	5.07	-3.78	
					Fraction < 100 μm	5.01	-3.78	- 11 ± 46 (modern)*
WP50A-1	N41°01.954'' W112°16.336''	Buried (-0.10)	Buried microbial mat	Flat microbial mat	Bulk	2.07	-5.99	
WP68A-1	N41°02.063'' W112°16.448''	0	Microbialite	Thrombolitic crust	Top	3.88	-3.30	
					Bottom	4.51	-4.41	
WP68A-4	N41°02.063'' W112°16.448''	0	Microbialite	Thrombolitic crust	Bulk	4.53	-4.71	
WP137A-1	N40°42.225 W112°14.969	1	Lithifying microbial mat	Microbial head				
WP342-3B	N41°01.669'' W112°16.354''	0.4	Microbialite	Column	Aragonite	4.98	-5.54	2811 ± 55
					Dolomite	3.06	-4.05	
WP346-1	N41°01.661 W112°16.396	0.5	Lithifying microbial mat	Pustular microbial mat				
WP366-1	N41°01.666'' W112°16.344''	0.04	Lithifying microbial mat	Column				
WP367-1	N41°01.659'' W112°16.385''	x	Lithifying microbial mat	Pustular microbial mat				
WP1007-1	N41°01.856''W 112° 16.575''	0.5	Lithifying microbial mat	Column	Bulk < 2μm			
WP1012-1	N°40.58.014 W112°15.809	0.5	Lithifying microbial mat	Column				

Supplementary Table S1: List of main characteristics of the samples analysed in this study.

	Number of spot	Aragonite	Mat green layer	Mat orange layer	Ooid substrat
High SR	46	45	1	0	0
Medium SR	31	20	3	2	6
Low SR	17	14	1	2	0
Total number of SR hotspots	94	79	5	4	6
	**% of superposition with SR**	84.04	5.32	4.26	6.38

Supplementary Table S2: Correspondence between sulfate reduction (SR) hotspots and aragonite patches in the lithifying mat (WP367-1; Fig. 1B).

A	Oxides (At%)	Lithifying mat (WP20A-1)		Microbialite (WP342-3B)		Standard Stevensite	Standard Sepiolite
		Av	SD	Av	SD		
	SiO2	52.36	1.88	61.38	1.81	51.2	69.04
	MgO	42.02	1.82	35.32	0.95	23.4	30.57
	Al2O3	3.55	3.11	0.53	0.29	1.6	0.25
	CaO	1.56	0.61	1.02	0.90	0.6	0.02
	Na2O	0.20	0.10	0.14	0.06	1.4	
	K2O	0.12	0.05	0.21	0.14	0.3	0.06
	MnO	0.09	0.06	1.07	0.46	1.6	
	Fe2O3	0.05	0.05	0.31	0.19		0.07
	TiO2	0.03	0.04	0.02	0.03		

B		Lithifying mat (WP20A-1)		Microbialite (WP342-3B)		Standard stevensite
		Av	SD	Av	SD	
	Si	3.28	0.11	3.77	0.08	3.92
	Mg	3.92	0.17	3.24	0.09	2.67
	Al	0.26	0.23	0.04	0.02	0.08
	Ca	0.10	0.04	0.07	0.06	0.05
	Na	0.02	0.01	0.02	0.01	0.21
	K	0.01	0.00	0.02	0.01	0.03
	Mn	0.00	0.00	0.06	0.02	0.10
	Fe	0.00	0.00	0.01	0.01	0.01
	Ti	0.00	0.00	0.00	0.00	0.00
	Tetra	3.54		3.81		4.00
	Octa	3.93		3.31		2.84
	Charge	0.24		0.17		

C		Lithifying mat (WP20A-1)		Microbialite (WP342-3B)		Standard sepiolite
		Av	SD	Av	SD	
	Si	9.53	0.33	10.97	0.24	11.98
	Mg	11.40	0.48	9.41	0.28	7.91
	Al	0.76	0.67	0.11	0.06	0.02
	Ca	0.30	0.12	0.20	0.18	0.00
	Na	0.07	0.04	0.05	0.02	0.00
	K	0.03	0.01	0.05	0.03	0.01
	Mn	0.01	0.01	0.16	0.07	0.00
	Fe	0.01	0.01	0.04	0.03	0.01
	Ti	0.00	0.01	0.00	0.00	0.00
	Tetra	10.29		11.08		12.00
	Octa	11.43		9.62		7.97
	Charge	0.71		0.49		0.02

Supplementary Table S3 (previous page): EDS of representative Mg-Si phases in a lithifying mat and a microbialite. (Av: average on 10 measurements; SD: standard deviation). B. Number of atoms normalized to 11 oxygen atoms compared with stevensite [3]. The theoretical structural formula, is calculated as tetrahedron (Tetra). C. Number of atoms normalized to 32 oxygen atoms compared with sepiolite [4]. The theoretical structural formula, is calculated as tetrahedron (Tetra) and octahedron (Octa).

	Latitude (NAD 27)	Longitude (NAD 27)	Depth (m)	pH	T°	Salinity (ppth)	Na^+ (mg/L)	Mg^{2+} (mg/L)	K^+ (mg/L)	Ca^{2+} (mg/L)	Cl^- (mg/L)	SO_4^{2-} (mg/L)	Si (mg/L)	Alkalinity (mg/L)
**May 2013**	N40°53'56"	W112°20'56"	0.20	8.20	13*	120	38000	4020	2390	226	72600	8560	7.65	380
**July 2013**	N40°53'56"	W112°20'56"	0.10	8.20	26*	129	38400	4220	2490	235	67800	8070	7.76	345

Supplementary Table S4: Chemical composition of GSL water at station GSL 3510 in May and July 2013 (http://www.usgs.gov/water/).

*Temperature data were unavailable, so they were replaced by June and August 2013 temperatures at the same location.

SI References

1. Pitzer, K. S. *Theory: Ion Interaction Approach, Chapter 7 in Activity Coefficients in Electrolyte Solutions, RM Pytkowicz, Ed*. (CRC Press, Boca Raton, FL, 1979).

2. Anderson, R. B. *et al.* Quantity and quality of groundwater discharge in a hypersaline lake environment. *J. Hydrol.* 512, 177–194 (2014).

3. Kasai, K., Sato, K., Kimura, S., Shakunaga, N. & Obara, K. Characterization of smectite scale and scale inhibition test by pH control at the Mori geothermal power plant, Japan. *Proc World Geotherm. Congr.* 1331–1336 (2000).

4. García-Romero, E. & Suárez, M. On the Chemical Composition of Sepiolite and Palygorskite. *Clays Clay Miner.* 58, 1–20 (2010).
